# Comprehensive analysis of cuproptosis-related lncRNAs signature to predict prognosis in bladder urothelial carcinoma

**DOI:** 10.1186/s12894-023-01292-9

**Published:** 2023-07-21

**Authors:** Shusheng Zhu, Houying Li, Yanpeng Fan, Chao Tang

**Affiliations:** 1 Department of Urology, Jining No. 1 People’s Hospital, Jining, shandong China; 2 Department of medical imaging center, The Second Hospital of Shandong University, Jinang, Shandong China; 3 Department of Urology, The First Hospital of Jilin University, Changchun, Jilin China; 4 Department of Urology, Affiliated Yantai Yuhuangding Hospital, Qingdao University, Yantai, 264000 Shandong China

**Keywords:** Bladder urothelial carcinoma, Cuproptosis, lncRNA, Risk model

## Abstract

**Background:**

Cuproptosis-related genes (CRGs) have been recently discovered to regulate the occurrence and development of various tumors by controlling cuproptosis, a novel type of copper ion-dependent cell death. Although cuproptosis is mediated by lipoylated tricarboxylic acid cycle proteins, the relationship between cuproptosis-related long noncoding RNAs (crlncRNAs) in bladder urothelial carcinoma (BLCA) and clinical outcomes, tumor microenvironment (TME) modification, and immunotherapy remains unknown. In this paper, we tried to discover the importance of lncRNAs for BLCA.

**Methods:**

The BLCA-related lncRNAs and clinical data were first obtained from The Cancer Genome Atlas (TCGA). CRGs were obtained through Coexpression, Cox regression and Lasso regression. Besides, a prognosis model was established for verification. Meanwhile, Kyoto Encyclopedia of Genes and Genomes (KEGG) enrichment analysis, gene ontology (GO) analysis, principal component analysis (PCA), half-maximal inhibitory concentration prediction (IC50), immune status and drug susceptibility analysis were carried out.

**Results:**

We identified 277 crlncRNAs and 16 survival-related lncRNAs. According to the 8-crlncRNA risk model, patients could be divided into high-risk group and low-risk group. Progression-Free-Survival (PFS), independent prognostic analysis, concordance index (C-index), receiver operating characteristic (ROC) curve and nomogram all confirmed the excellent predictive capability of the 8-lncRNA risk model for BLCA. During gene mutation burden survival analysis, noticeable differences were observed in high- and low-risk patients. We also found that the two groups of patients might respond differently to immune targets and anti-tumor drugs.

**Conclusion:**

The nomogram with 8-lncRNA may help guide treatment of BLCA. More clinical studies are necessary to verify the nomogram.

**Supplementary Information:**

The online version contains supplementary material available at 10.1186/s12894-023-01292-9.

## Introduction

Bladder urothelial carcinoma (BLCA) is a global health concern and ranks among the top 10 most prevalent types of cancer, with an annual incidence of 550,000 new cases and a mortality rate of 200,000 [1, 2]. Among the 80% of patients with non-muscle-invasive bladder cancer, about 50% of patients relapse after surgery [3, 4]. Due to the absence of specific biomarkers, a portion of severe BLCA patients still cannot receive effective treatment. Although BLCA can be treated surgically in the early stages, the prognosis of advanced BLCA is poor. Innovative therapies and prognostic models are necessary to enhance the prognosis of advanced BLCA.

Metal micronutrients, especially copper, iron, and zinc are essential for life [5]. Copper plays a crucial role as a coenzyme in fundamental enzymes, such as those involved in mitochondrial respiration, immune system function, and the elimination of free radicals [6–8]. A recent report showed that the level of copper in the serum and tumor tissues of cancer patients was significantly increased, and copper could be directly combined with the fatty components of the TCA cycle [9–11]. Tsvetkov et al. found a new mechanism of copper-induced cell death related to mitochondrial dysfunction [12], this new form of controlled cell death was named “cuproptosis”. Cuproptosis might happen when mitochondrial enzymes aggregate and lead to mitochondrial stress [13]. This indicates that cuproptosis could potentially serve as a latent target for immunotherapy in BLCA.

Long noncoding RNAs (lncRNAs) are a class of RNA molecules that exceed 200 nucleotides in length and lack protein-coding capacity [14]. As a new class of cellular regulatory molecules, lncRNAs interact with various molecules, depending on their subcellular distribution, to modulate gene transcription and kinase cascades [15]. Meanwhile, lncRNAs participate in mRNA expression and gene regulation, affecting various aspects of tumor cells [16].

In this paper, we investigated the potential involvement of crlncRNAs in the prognosis of BLCA patients. The results could aid in predicting the prognosis of BLCA patients and identifying potential drugs.

## Materials and methods

### Download and processing of TCGA data

We retrieved RNA sequencing (RNA-seq) transcription data, expression and mutation files, as well as clinical information of BLCA patients from the Cancer Genome Atlas (TCGA) database (https://portal.gdc.cancer.gov/). The data included tumor tissues of 412 BLCA samples and 19 normal tissue samples. All data were acquired and processed in accordance with the guidelines set forth by TCGA.

### Selection and differential expression analysis of crlncRNAs

According to the research of Tsvetkov et al. [12], we selected 19 CRGs (**Additional File Table**S1) for analysis. Then, the correlation between CRGs and differentially expressed lncRNAs was analyzed. All 277 crlncRNAs met the criteria of Pearson’s correlation coefficient (|PearsonR|) > 0.5 and *p* < 0.001.

### CrlncRNAs risk signature for BLCA prognosis

Univariate Cox regression analysis was performed on the obtained clinical and demographic data of BLCA patients to determine the lncRNA related to the overall survival (OS) rate of BLCA patients. On the basis of 10-fold cross-validation, Lasso regression was conducted to screen the lncRNAs that were really related to the survival of patients. Meanwhile, 8 optimal lncRNAs related to BLCA prognosis were screened and used to establish the risk model. Risk score $={\sum }_{i=1}^{n}\text{E}\text{x}\text{p} \left(\text{l}\text{n}\text{c}\text{R}\text{N}\text{A}\right) \times \text{c}\text{o}\text{e}\text{f} \left(\text{l}\text{n}\text{c}\text{R}\text{N}\text{A}\right)$. Where coef (lncRNA) was the regression coefficient and $\text{E}\text{x}\text{p}$(lncRNA) was the expression level of crlncRNAs.

We randomly divided 277 BLCA samples into train group and test group in 1:1 ratio, and classified BLCA patients into high-risk and low-risk groups [17, 18]. The prognostic characteristics were analyzed using the Chi-square test, Receiver operating characteristic (ROC) curves analysis, and Kaplan-Meier survival analysis. We further made the nomogram with crlncRNAs risk score, established clinical risk factors, and predicted the survival time of patients. Then, the concordance index (C-index) and calibration curves of a nomogram model were built to investigate the prediction power of the nomogram. Finally, we carried out a stratified analysis to assess whether this feature maintained its predictive power in patient subgroups (phases I – II and III – IV).

### Principal component analysis, gene ontology and functional enrichment analysis

Principal component analysis (PCA) was performed to describe the expression model of crlncRNAs. At the same time, the relationship between the three variables of the sample was visualized with 3D scatter plots. Then, the analysis of differentially expressed genes (DEGs) was performed [(Log2 fold change (FC) > 1, fdrFilter (FDR) < 0.05]. The Gene Ontology (GO) was used to elucidate the DEGs in terms of relevant cellular components (CC), biological processes (BP), and molecular functions (MF). Gene Set Enrichment Analysis (GSEA) was used to conduct differential Kyoto Encyclopedia of Genes and Genomes (KEGG) pathways, with a false discovery rate (FDR) less than 0.25.

### Investigation of tumor immune function and mutation burden

We used Single-sample GSEA (ssGSEA) enrichment score to describe which genes were coordinately upregulated or downregulated within a sample. Meanwhile, we obtained the somatic mutation file and calculated the tumor mutation burden (TMB) score for each BLCA patient. Kaplan Meier analysis was used to evaluate the impact of TMB on BLCA patients’ OS, and a t-test was used to compare the difference between high-risk groups and low-risk groups. Then, the Tumor Immune Dysfunction and Exclusion (TIDE) model was utilized to forecast immune response by simulating tumor immune escape mechanisms (http://tide.dfci.harvard.edu) [19, 20].

### Drug sensitivity prediction

Inhibitory concentrations (IC50) represented the semiinhibitory concentration of the measured antagonist [20]. To assess drug candidates for BLCA treatment in clinical trials, we utilized pRRophetic to calculate the half-maximal IC50 values [13, 20, 21].

## Statistical analysis

We utilized the R software (version 4.1.3) for conducting scientific statistical analyses. The “limma” package was used to combine RNA-seq transcriptome and somatic mutation data. Pearson correlation test was used to obtain crlncRNAs. Then, cox regression, survival, univariate and multivariate analysis were conducted. Besides, in order to evaluate the predictive performance of the risk model, time-dependent ROC curve analysis was performed via “survivalROC” R package. The GO and KEGG were analyzed using the “clusterProfiler” package in R software. Wilcoxon rank-sum test and chi-square test were used to compare the difference between two groups. The OS time of the two groups was estimated using Kaplan-Meier analysis with a log-rank test, and statistical significance was indicated by p < 0.05 for all analyses.

## Results

### Identification of crIncRNA

**Fig. 1:**
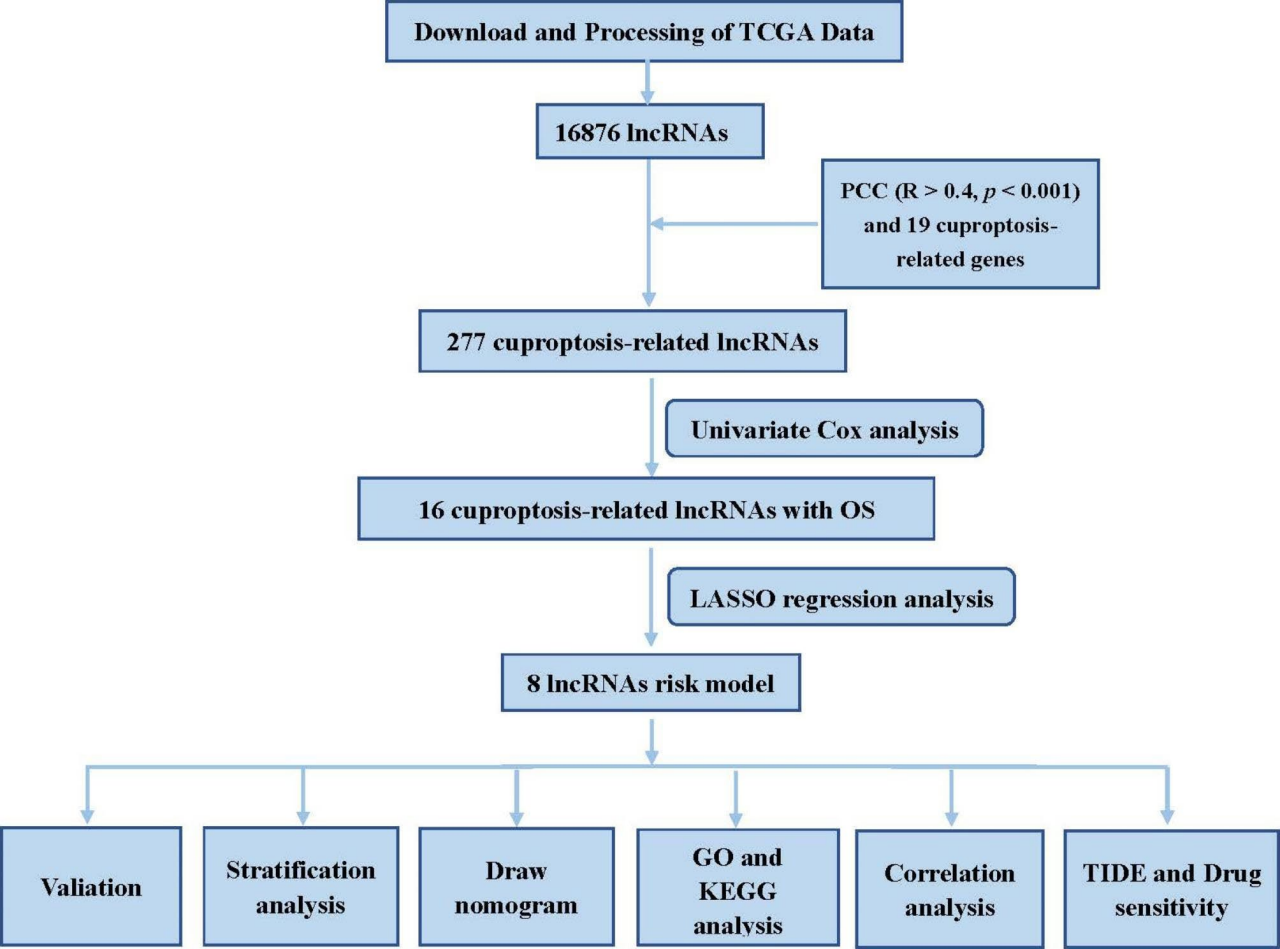
Flowchart of the present research. TCGA, The Cancer Genome Atlas; lncRNAs, long noncoding RNAs; PCC, pearson’s correlation coefficient; OS: overall survival; GO, gene ontology; KEGG, Kyoto Encyclopedia of Genes and Genomes

**Fig. 2:**
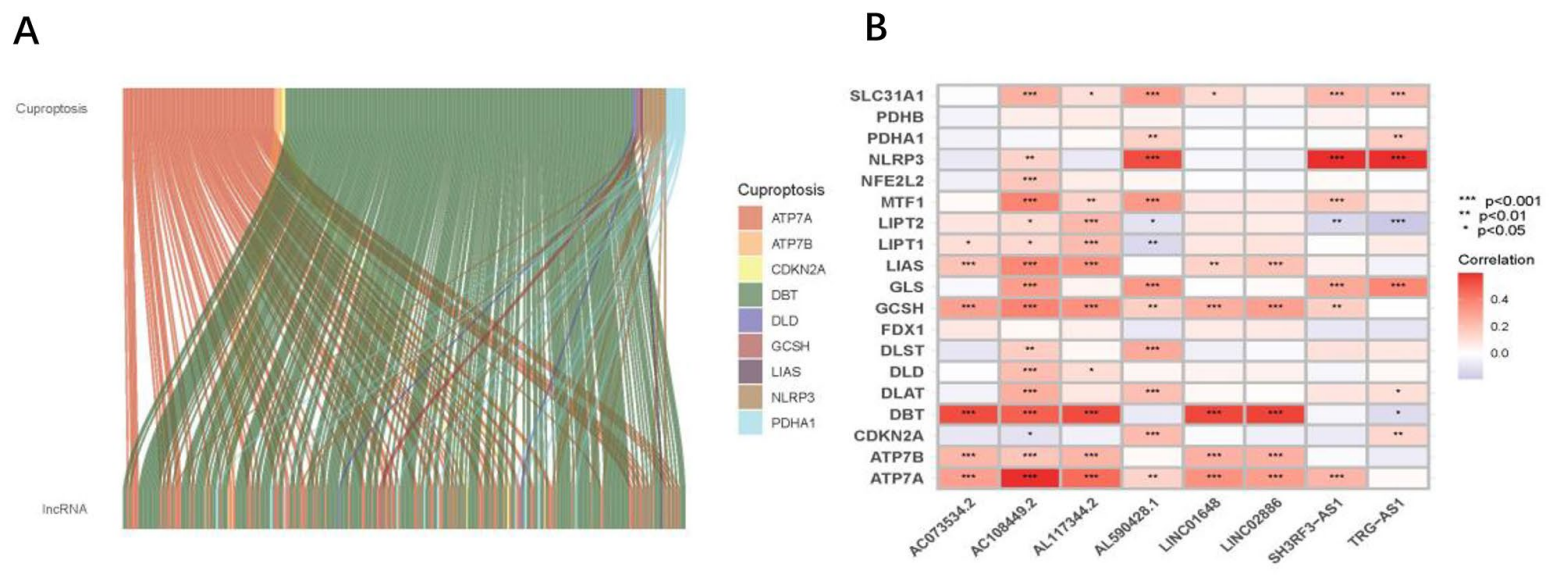
(**A**) Sankey diagram of coexpression between 19 CRGs and 277 crlncRNAs. (**B**) correlation 19 CRGs and 8 prognostic crlncRNAs. CRGs, cuproptosis-related genes; crlncRNAs, cuproptosis-related long noncoding RNAs

The design route for analysis can be seen in Fig. 1. We identified 16,876 lncRNAs in the TCGA_BLCA dataset. In total, we obtained 19 CRGs. Based on Pearson analysis, 277 crlncRNAs were found. We made sankey plot and heat map to show the association between CRGs and crlncRNAs (Fig. 2A, B). The 403 patients were divided into the train group (n = 202) and the test group (n = 201), and the clinical information on BLCA was presented in Table 1. The results showed that there was no difference between the train and test groups in all clinical features.

**Table 1:** Characteristic of bladder urothelial carcinoma patients

Covariates	Type	Total	Test	Train	Pvalue
Age	≤ 65	159(39.45%)	78(38.81%)	81(40.1%)	0.870
	> 65	244(60.55%)	123(61.19%)	121(59.9%)	
Gender	FEMALE	106(26.3%)	51(25.37%)	55(27.23%)	0.757
	MALE	297(73.7%)	150(74.63%)	147(72.77%)	
Grade	High Grade	380(94.29%)	189(94.03%)	191(94.55%)	1.000
	Low Grade	20(4.96%)	10(4.98%)	10(4.95%)	
	unknow	3(0.74%)	2(1%)	1(0.5%)	
Stage	Stage I	2(0.5%)	1(0.5%)	1(0.5%)	0.867
	Stage II	127(31.51%)	63(31.34%)	64(31.68%)	
	Stage III	140(34.74%)	73(36.32%)	67(33.17%)	
	Stage IV	132(32.75%)	62(30.85%)	70(34.65%)	
	unknow	2(0.5%)	2(1%)	0(0%)	
T	T1	3(0.74%)	2(1%)	1(0.5%)	0.927
	T2	117(29.03%)	60(29.85%)	57(28.22%)	
	T3	193(47.89%)	95(47.26%)	98(48.51%)	
	T4	57(14.14%)	29(14.43%)	28(13.86%)	
	unknow	33(8.19%)	15(7.46%)	18(8.91%)	
M	M0	194(48.14%)	100(49.75%)	94(46.53%)	1.000
	M1	11(2.73%)	6(2.99%)	5(2.48%)	
	unknow	198(49.13%)	95(47.26%)	103(50.99%)	
N	N0	234(58.06%)	123(61.19%)	111(54.95%)	0.239
	N1	46(11.41%)	25(12.44%)	21(10.4%)	
	N2	75(18.61%)	34(16.92%)	41(20.3%)	
	N3	6(1.49%)	1(0.5%)	5(2.48%)	
	unknow	42(10.42%)	18(8.96%)	24(11.88%)	

### Construction and validation of the crlncRNAs risk model

**Fig. 3:**
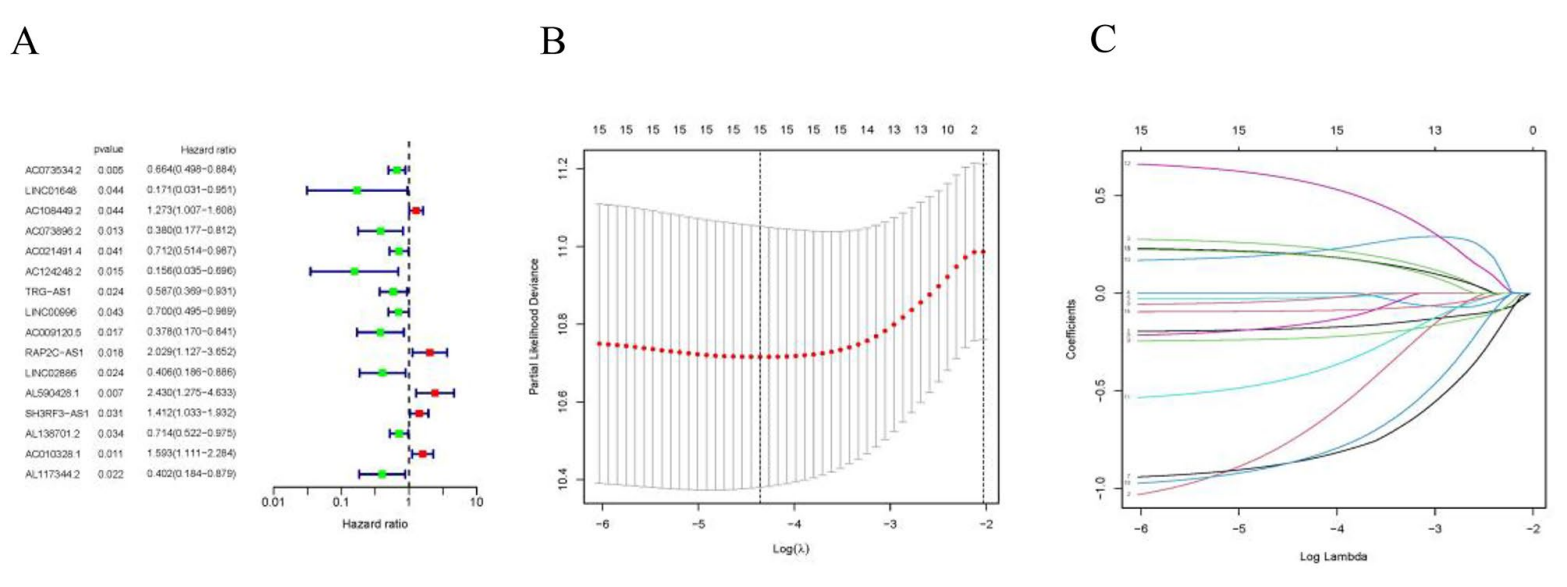
Identification of crlncRNAs with prognostic value in BLCA patients. (**A**) univariate Cox regression analysis for identifying the prognostic crlncRNAs. (**B**–**C**) Lasso–Cox regression analysis was conducted to construct prognostic prediction models. BLCA, bladder urothelial carcinoma; crlncRNAs, cuproptosis-related long noncoding RNAs

**Fig. 4:**
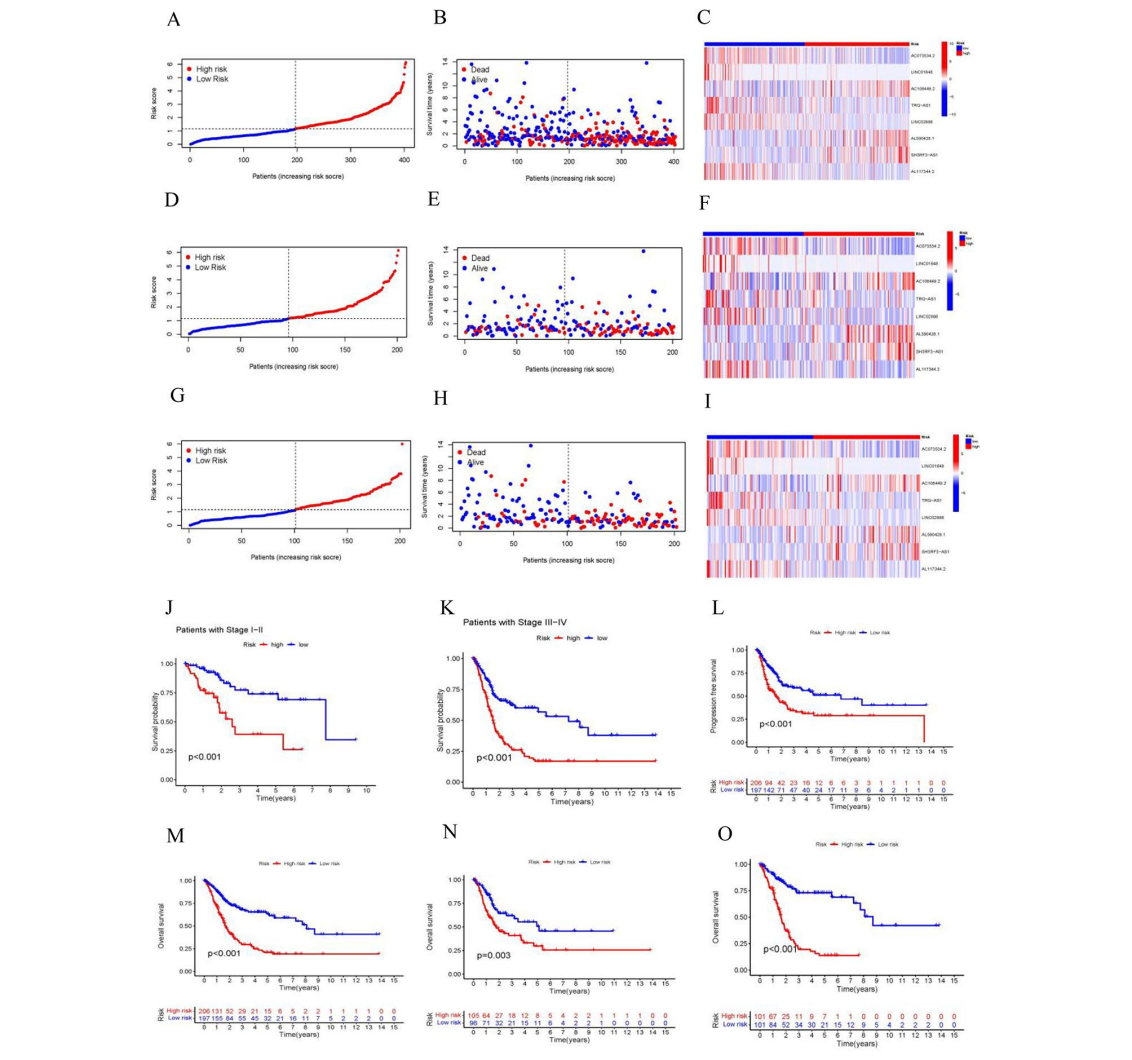
Prognosis capability of the model in the three patient sets. (**A**-**C**)Risk score distribution, survival status and heatmap for BLCA patients in high-risk and low-risk groups in the entire cohort. (**D**-**F** Risk score distribution, survival status and heatmap for BLCA patients in high-risk and low-risk groups in the test cohort. (**G**-**I** Risk score distribution, survival status and heatmap for BLCA patients in high-risk and low-risk groups in the train cohort.(**J**-**K**) Stage I-II and Stage III-IV survival analysis in the high- and low-risk groups. (**L**) Kaplan–Meier curves of progression-free survival. (**M**-**O**) Kaplan–Meier curves for overall survival analysis in the entire, test and train cohort. BLCA, bladder urothelial carcinoma

Univariate Cox regression analysis identified 16 lncRNAs as prognostic factors for the survival of BLCA patients **(**Fig. 3A**).** To avoid overfitting, LASSO regression method was performed **(**Fig. 3B, C**)**. Then, multiple Cox regression analysis was conducted, and we selected eight crlncRNAs prognostic markers to establish the risk model. Risk score = EXP_AC073534.2_ × (-0.268) + EXP_LINC01648_ × (-1.317) + EXP_AC108449.2_ × (0.321) + EXP_TRG−AS1_ × (-1.102) + EXP_LINC02886_ × (-0.702) + EXP_AL590428.1_ × (0.686) + EXP_SH3RF3−AS1_ × (0.306) + EXP_AL117344.2_ × (-1.047). Then, BLCA patients were categorized into low-risk and high-risk groups based on the median value of their risk score. The risk-score distribution plot revealed a negative correlation between survival times and increasing risk-scores (Fig. 4A-I). The analysis results showed that the high-risk group had worse survival (Fig. 4J-O).

### Evaluation of the risk model

**Fig. 5:**
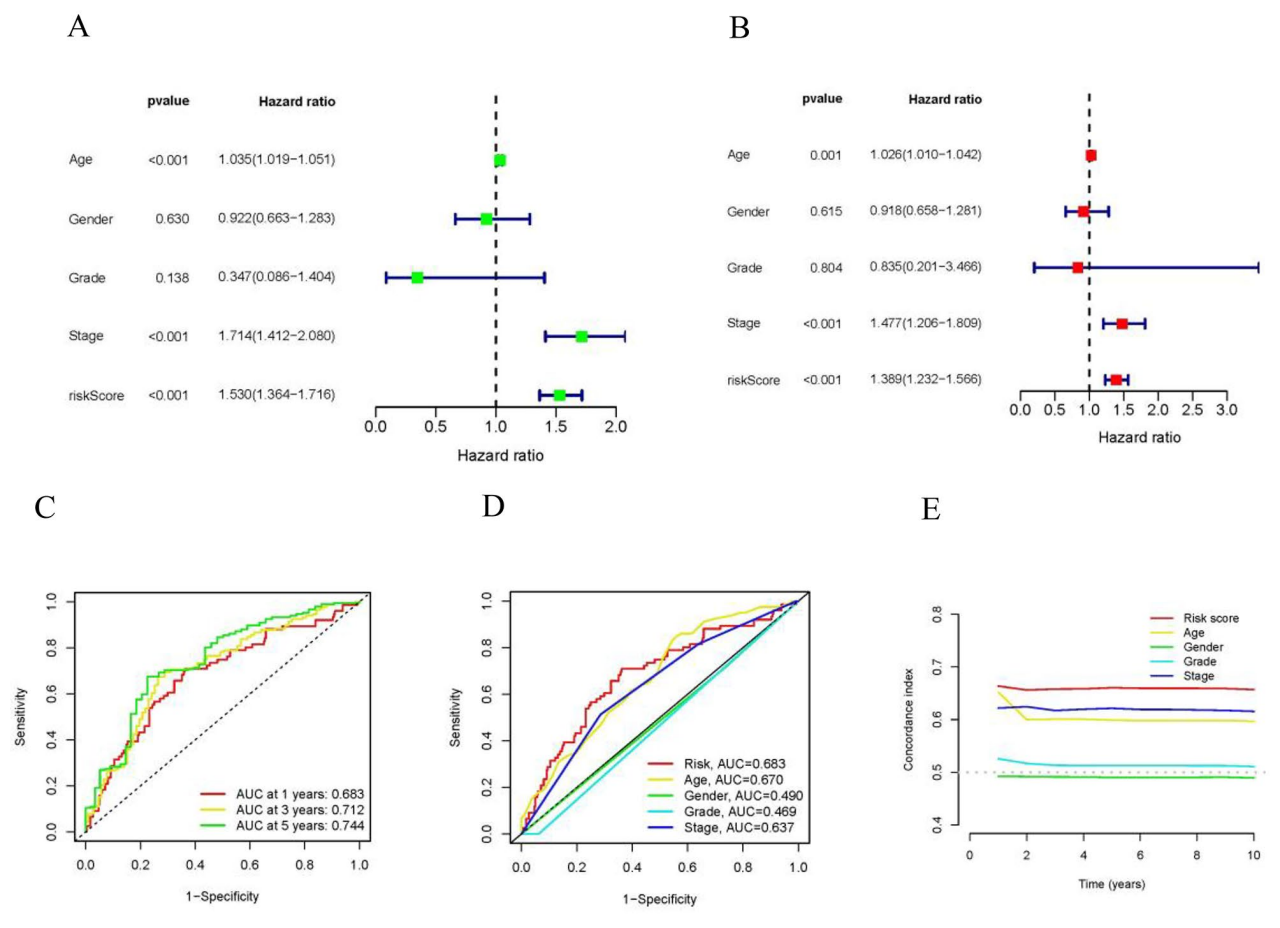
Independent prognostic analysis of BLCA OS. (**A**-**B**) Forest plot for univariate and multivariate Cox regression analysis. (**C**) Time-ROC curves predicted 1, 3, and 5-year of OS for BLCA patients. (**D**-**E**) ROC curves and C-index curves showed the predictive accuracy of the risk model was superior to other clinical factors. BLCA, bladder urothelial carcinoma; OS, overall survival; ROC, receiver operating characteristic

We utilized univariate and multivariate Cox regression analyses to assess the prognostic model’s predictive value. The age, risk score and stage were identified as significant independent prognostic factors **(**Fig. 5A, B**)**. The areas under the l-year, 3 years and 5 years ROC curves (AUC) were 0.683, 0.712, and 0.744 respectively **(**Fig. 5C**)**. The c-index and ROC curve results demonstrate that the accuracy of the prognosis model surpasses other clinical factors **(**Fig. 5D, E**)**.

### Construction of nomogram

**Fig. 6:**
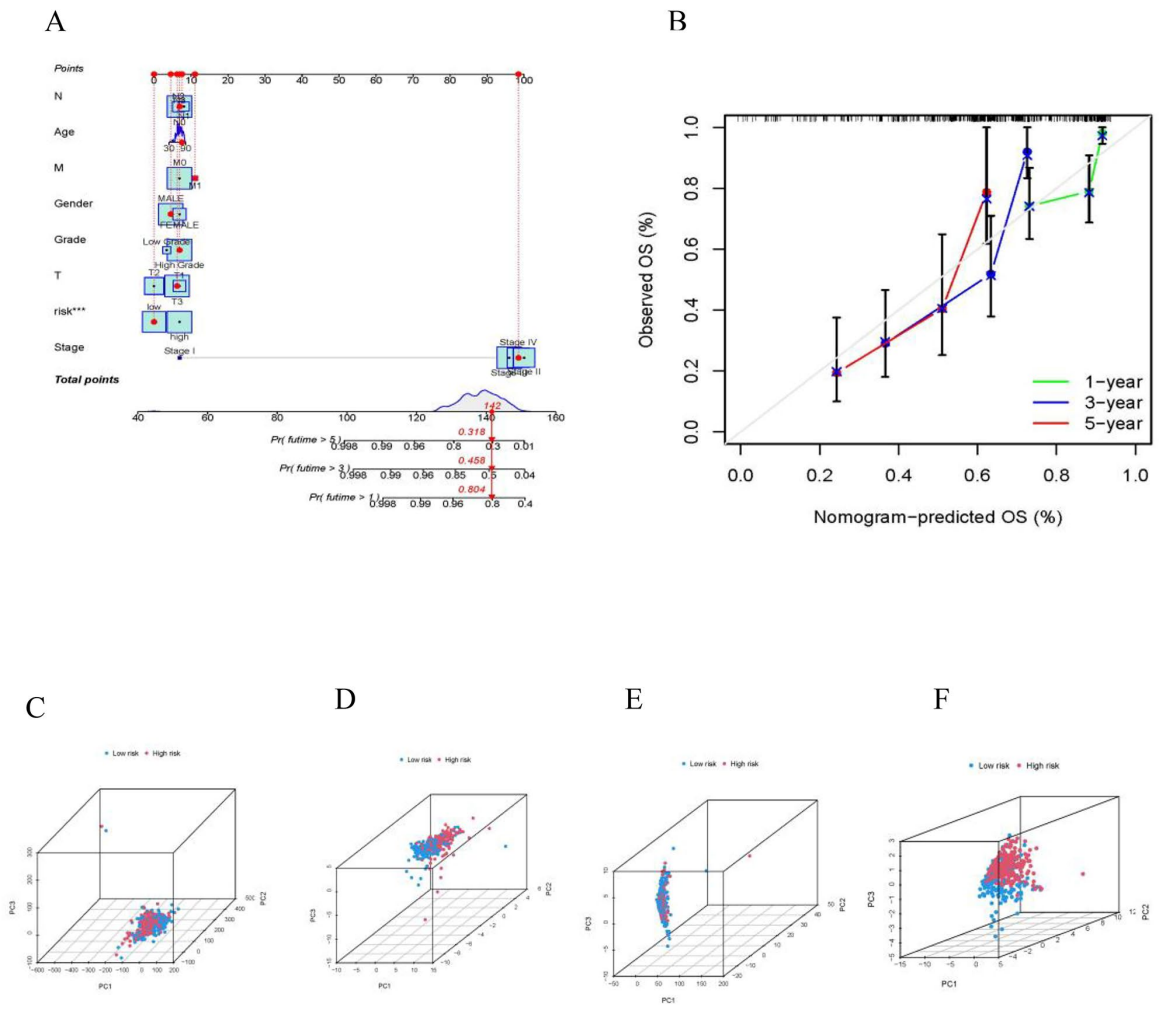
Nomogram for survival prediction (**A**) Nomogram was constructed based on crlncRNAs prognostic markers (**B**) Calibration plots for 1-, 3-, and 5-years survival predictions. (**C**-**F**) PCA between the high- and low-risk groups based on the (**C**) all genes, (**D**)CRGs, (**E**) crlncRNAs, and (**F**) crlncRNAs prognostic marker. PCA indicated differences in both risk groups and clusters. PCA, principal component analysis; crlncRNAs, cuproptosis-related long noncoding RNAs; CRGs cuproptosis-related genes; OS: overall survival

A nomogram model was developed to accurately predict the overall survival of patients with BLCA **(**Fig. 6A**)**. The calibration plots indicated good conformity with the prediction of this nomogram **(**Fig. 6B**)**. Afterwards, the 3D scatter diagram revealed distinct aggregation features of PCA in both low- and high-risk groups **(**Fig. 6C, D, E, F**)**.

### Functional and pathway analysis

**Fig. 7:**
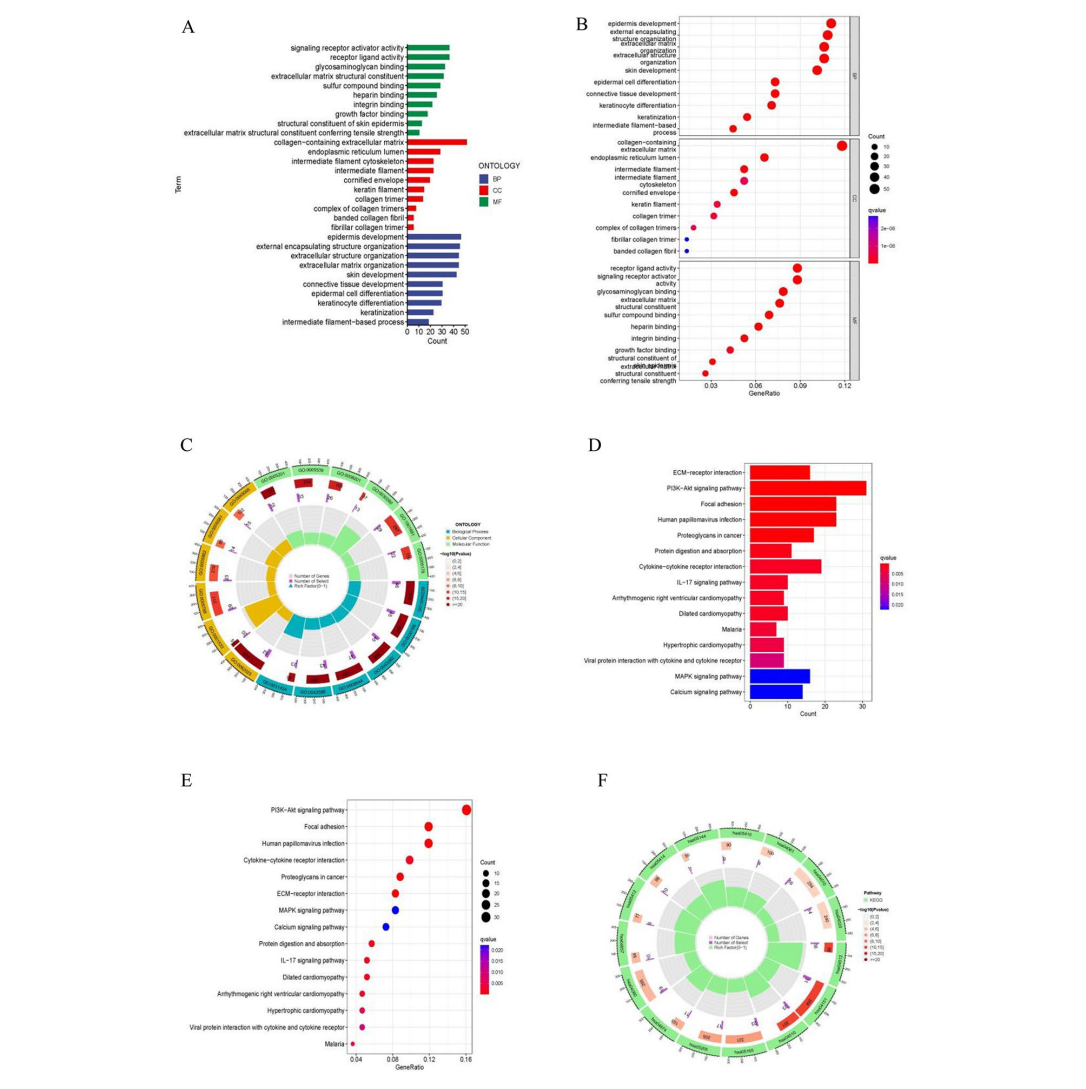
GO and KEGG pathway enrichment analysis. (**A**-**C**) Barplot, bubble chart and circle diagram of the GO enrichment terms. (**D**-**F**) Barplot, bubble chart and circle diagram of the KEGG enrichment terms. GO, gene ontology; KEGG, Kyoto Encyclopedia of Genes and Genomes

We further conducted the GO and KEGG enrichment analyses to find the biological functions and pathway analysis of DEGs. A total of 608 DEGs were obtained between two groups (**Additional File Table**S2). In the biological process category, the genes were mainly associated with signaling receptor activator activity, receptor ligand activity and glycosaminoglycan binding. In the cellular component category, it was mainly enriched in the collagen − containing extracellular matrix, endoplasmic reticulum lumen and intermediate filament cytoskeleton. In the molecule function category, it was epidermis development, external encapsulating structure organization and extracellular structure organization **(**Fig. 7A, B, C**)**. Genes in the KEGG [22–24] category were enriched in the PI3K − Akt signaling pathway, focal adhesion and human papillomavirus infection **(**Fig. 7D, E, F**)**.

### Immunity analysis of the risk score and tumor mutational burden

**Fig. 8:**
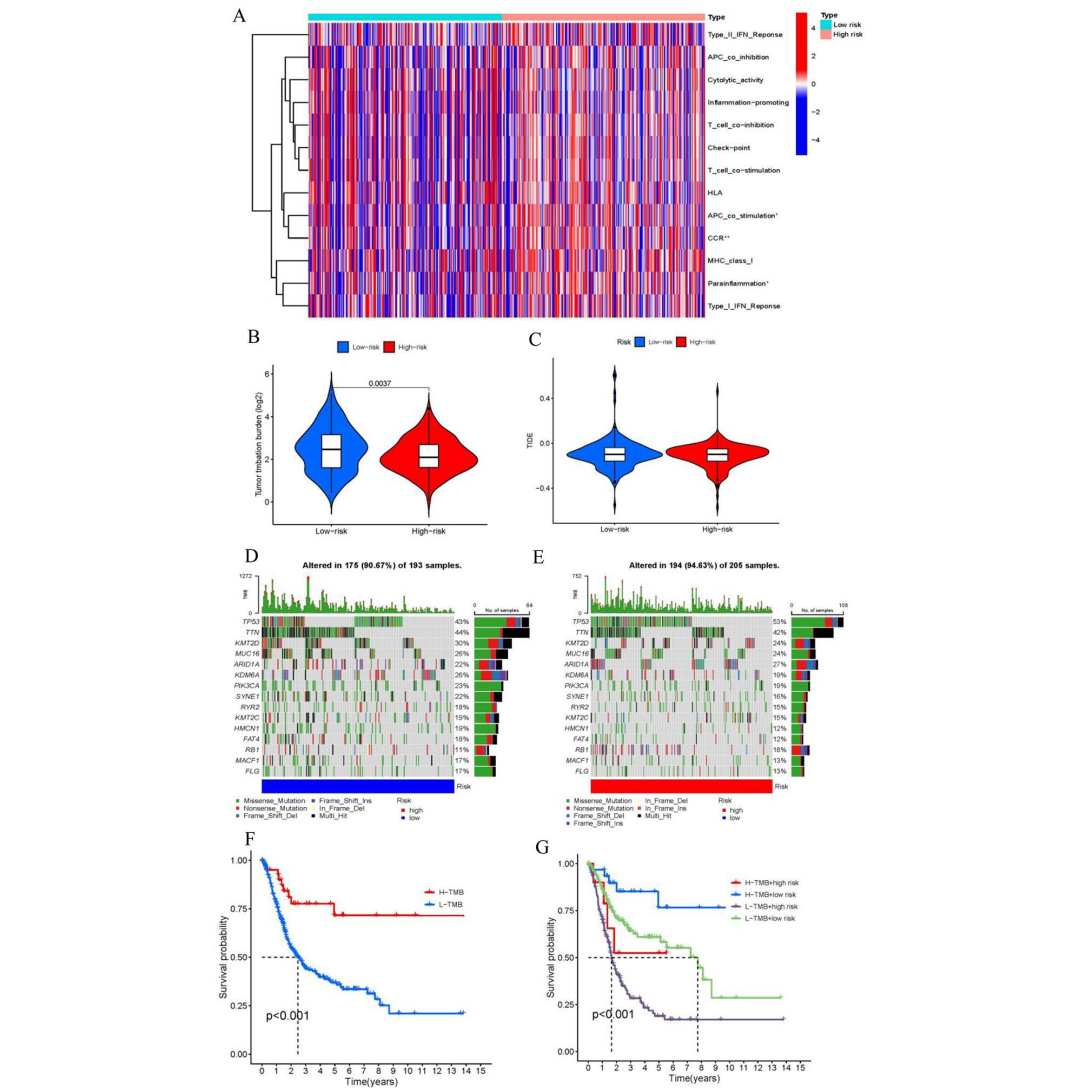
Immunological landscape in BLCA patients and relationship between TMB and risk scores. (**A**) Heatmap of the tumor-infiltrating lymphocytes among two groups in BLCA. (**B**-**C**) Analysis of TMB differences and TIDE prediction score. (**D**-**E**) Waterfall plot of top 15 mutation genes in the high-risk and low-risk group in BLCA. (**F**) Overall survival analysis curves of the high- and low-TMB groups. (**G**) Overall survival curve combined with TMB risk in BLCA. TMB, tumor mutation burden; TIDE, tumor immune dysfunction and exclusion; BLCA, bladder urothelial carcinoma

Figure 8 A displays the immune response heatmap generated by the ssGSEA algorithm. According to ssGSEA analysis of TCGA-BLCA data, the correlation between immune cell populations and related functions showed significant differences in IFN response types, T cell functions, antigen-presenting cell (APC) functions, human leukocyte antigen (HLA), chemokine receptor (CCR), and inflammation between the two groups. We collected somatic mutation data in BLCA and TIDE data (http://tide.dfci.harvard.edu/), and then calculated the corresponding TMB and TIDE scores **(**Fig. 8B, C**)**. The results indicated that the high-risk group in BLCA had a greater burden of mutations compared to the low-risk group. We categorized BLCA patients into two groups, “High-TMB” and “Low-TMB,“ in order to conduct survival analyses. The fifteen most mutated genes were TP53, TTN, KMT2D, MUC16, ARID1A, KDM6A, PIK3CA, SYNE1, RYR2, KMT2C, HMCN1, FAT4, RB1, MACF1 and FLG. TP53 was the gene with the highest mutation frequency **(**Fig. 8D, E**)**. The results indicated that the low-TMB group exhibited a lower survival rate compared to the high-TMB group in BLCA **(**Fig. 8F**)**. Patients with lower scores and higher tumor mutational burden (TMB) exhibited the most favorable prognosis among the four groups **(**Fig. 8G**)**.

### Drug sensitivity analysis

**Fig. 9:**
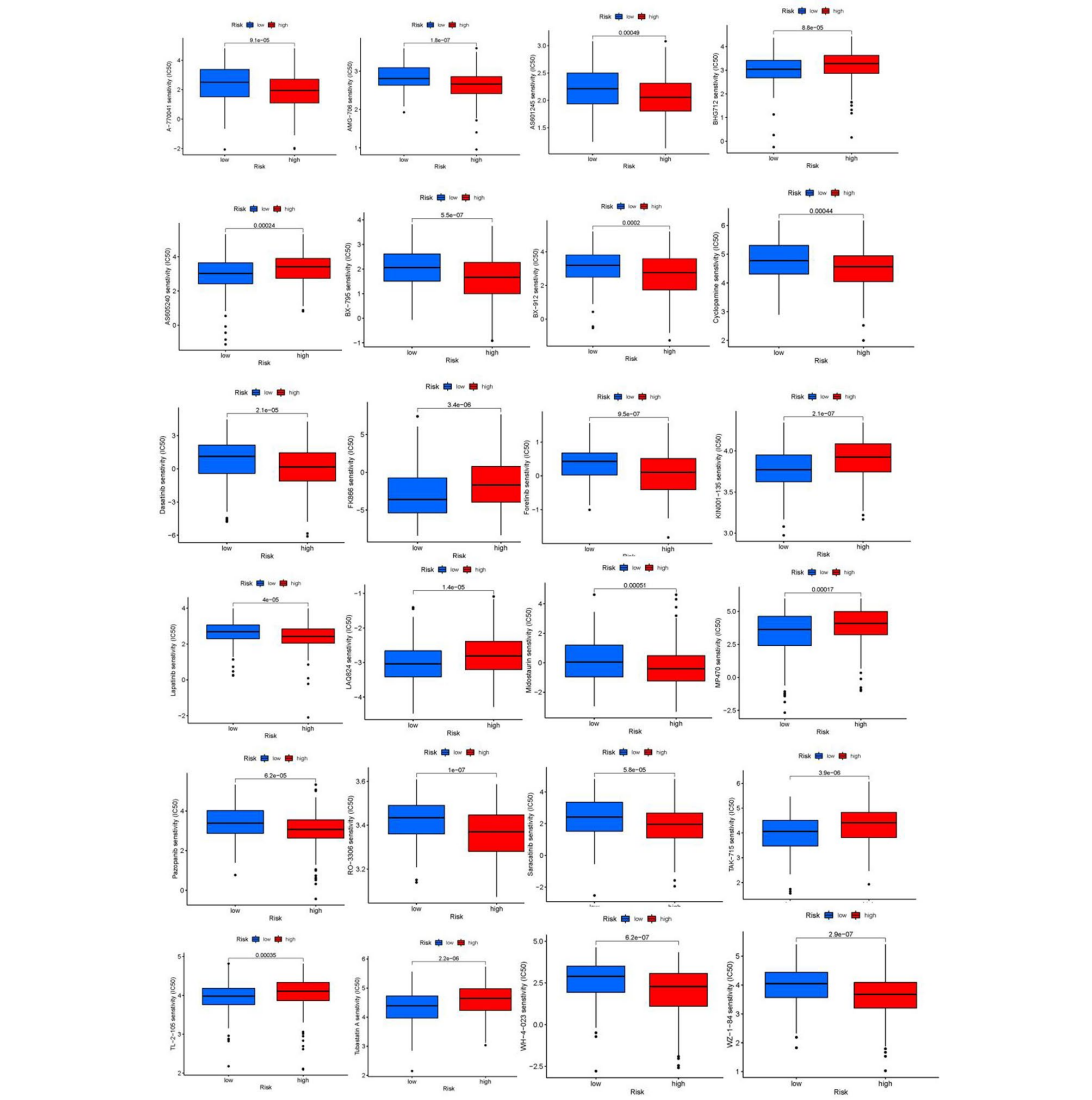
Drug sensitivity (IC50) correlated with high- and low-risk patients in bladder urothelial carcinoma. IC50, half-maximal inhibitory concentration prediction

We aimed to investigate the potential of crlncRNAs as prognostic markers for personalized treatment of BLCA by examining the correlation between drug risk scores and IC50 values in BLCA therapy. As shown in Fig. 9, the sensitivity of 24 anticancer drugs was significantly different in two groups (p < 0.05). These drugs have the potential to be used in the future for treating BLCA. The relationship between the risk scores and IC50 was shown in **Additional File Figure**S1.

## Discussion

Bladder urothelial carcinoma (BLCA) is a malignant tumor with a dismay outcome [25]. Due to complex pathological subtypes, genomic differences and drug resistance, the overall chemotherapy effect of BLCA is not very ideal [26]. It has been reported that an elevated level of copper in tumor patients can stimulate the growth of new blood vessels, facilitate the progression of tumors, and promote their spread to other parts of the body. Recently, Tsvetkov et al. have reported on the phenomenon of cuproptosis, which has been shown to overcome malignant cell resistance to chemotherapy and facilitate the removal of defective cells [12]. In addition, compared with normal cells, copper ionophores have inherent selectivity in inducing cancer cell clusters [20]. Therefore, cuproptosis may be a promising approach for treating BLCA in the future. Additionally, lncRNA exerts biological effects on the development and treatment of various cancers [20, 27, 28]. LncRNAs have been identified as significant prognostic factors in BLCA and may serve as promising molecular targets for its treatment [29–31]. The aim of this article is to identify potential prognostic markers by examining the possible interaction between lncRNA and cuproptosis.

In our research, we identified 16 crlncRNAs that can be used as prognostic markers for predicting the overall survival of patients with BLCA. Among them, eight were selected to construct a prognostic model. First, 19 CRGs and 277 crlncRNAs were identified. Then, Lasso regression and COX regression analyses were performed to identify the prognostic crlncRNAs. Different types of predictive lncRNA signatures have been reported in previous studies for patients with BCAL [4]. The highest AUC of the crlncRNAs signature in 5 years was 0.653 in the study by Zhang. In our study, the AUC in 5 years is 0.744, which demonstrates this crlncRNAs marker has strong predictive power. What’s more, we further identified the crlncRNAs clinical variables, immune cell infiltration and immunotherapy, and drug sensitivity of BLCA.

Based on Cox and Lasso regression analyses, we identified eight lncRNAs that are associated with prognosis: AC073534.2, LINC01648, AC108449.2, TRG-AS1, LINC02886, AL590428.1, SH3RF3-AS1 and AL117344.2. Ding et al. found that AC073534.2 was a prognostic biomarker for acute myeloid leukaemia [32]. LINC01648 was associated with glycosylated hemoglobin [33]. Sun et al. found that the expression level of AC108449.2 was correlated with the OS of BLCA patients. The expression level of AC108449.2 positively correlates with the risk score in BLCA [34]. TRG-AS1 has been reported to play an important role in many tumors [35–38]. One study showed that in pterygium fibroblasts, the expression of AL590428.1 was decreased [39]. SH3RF3-AS1 was found to be significant expression in hepatocellular carcinoma with cirrhosis [40]. However, there are few reports on LINC02886 and AL117344.2. Thus, it is imperative to further elucidate their mechanisms in future research.

Then, the accuracy of crlncRNAs prognostic model was verified. Based on the Kaplan Meier method, our study revealed that the high-risk group had a lower overall survival rate compared to the low-risk group. The ROC curves demonstrated that the prognostic model based on crlncRNAs exhibited high accuracy in predicting 1-, 3-, and 5-year survival rates, with all AUC values exceeding 0.65. The application of PCA also revealed the distinction between the two groups. By combining crlncRNAs prognostic model with clinical information, a nomogram was developed to predict prognosis and metastasis in BLCA patients using PFS and C-index.

The GO and KEGG analysis revealed that the differentially expressed crlncRNAs prognostic marker was significantly enriched in the PI3K-Akt signaling pathway, focal adhesion, and human papillomavirus infection. The PI3K/AKT signaling pathway governs cellular survival and proliferation. Abnormal activation of this pathway is typically linked to the progression of cancer and resistance to tumor therapies [13, 41]. Thus, further studies are needed to explore the relationship between cuproptosis and the PI3K/AKT signaling pathway. Waterfall plot revealed that TP53 was mutated more frequently in BLCA patients. Chemical damage induced by certain mutagens may be responsible for TP53 mutations [42]. We believed that elevated levels of copper in patients with bladder cancer may trigger TP53 mutations, which could be associated with the occurrence of cuproptosis. We utilized pRRophetic to predict the response of drugs in treating BLCA [21]. There were significant variations in IC50 values among the 24 drugs for high-risk and low-risk patients.

Cuproptosis is a novel form of programmed cell death that has the potential to become a significant therapeutic target in cancer treatment. Our study identified several crlncRNAs that play a significant role in cancer progression and treatment through various biological mechanisms. However, the current study had several limitations. First, we built and validated the prediction model with only a single retrospective BLCA data source. Second, there are still many unexplored areas between cuproptosis and lncRNAs. Thus, more validation is needed through preclinical studies. Third, since our scoring model requires the detection of the relevant genes, its applicability in clinical practice may lead to an increase in the cost-related burden for BLCA.

## Conclusion

In summary, we identified a risk model based on crlncRNAs expression to predict survival of patients with BLCA. Our study provided a novel therapeutic approach for personalized treatment and improved immunotherapy response in patients with BLCA. Meanwhile, the usefulness of nomogram in predicting BLCA patient survival needs to be explored in future studies.

## Electronic supplementary material

Below is the link to the electronic supplementary material.

Additional File Figure S1: Scatter plot between risk score and drug sensitivity.

Additional File Table S1: 19 cuproptosis-related genes.

Additional File Table S2: Data of differentially expressed genes.

## Data Availability

The datasets analysed during the current study are available in the TCGA database (https://portal.gdc.cancer.gov/). The original contributions presented in the study are included in the article/Supplementary Material, further inquiries can be directed to the corresponding authors.

## Abbreviations

CRGs: Cuproptosis-related genes
crlncRNAs: Cuproptosis-related long noncoding RNAs
BLCA: Bladder urothelial carcinoma
TME: Tumor microenvironment
TCGA: The Cancer Genome Atlas
KEGG: Kyoto Encyclopedia of Genes and Genomes
GO: Gene ontology
PCA: Principal component analysis
IC50: Half-maximal inhibitory concentration prediction
PFS: Progression-Free-Survival
C-index: Concordance index
OS: Overall survival
ROC: Receiver operating characteristic
DEGs: Differentially expressed genes
CC: Cellular components
BP: Biological processes
MF: Molecular functions
GSEA: Gene Set Enrichment Analysis
ssGSEA: Single-sample GSEA
TMB: Tumor mutation burden
TIDE: Tumor immune dysfunction and exclusion
RNA-seq: RNA sequencing
APC: Antigen-presenting cell
HLA: Human leukocyte antigen
CCR: Chemokine receptor
PCC: Pearson’s correlation coefficient
