# Supplementary figures and images for: Comprehensive analysis of cuproptosis-related lncRNAs signature to predict prognosis in bladder urothelial carcinoma
Source: BMC Urol. 2023 Jul 21;23:124. doi: 10.1186/s12894-023-01292-9 (PMC10362680; doi:10.1186/s12894-023-01292-9)

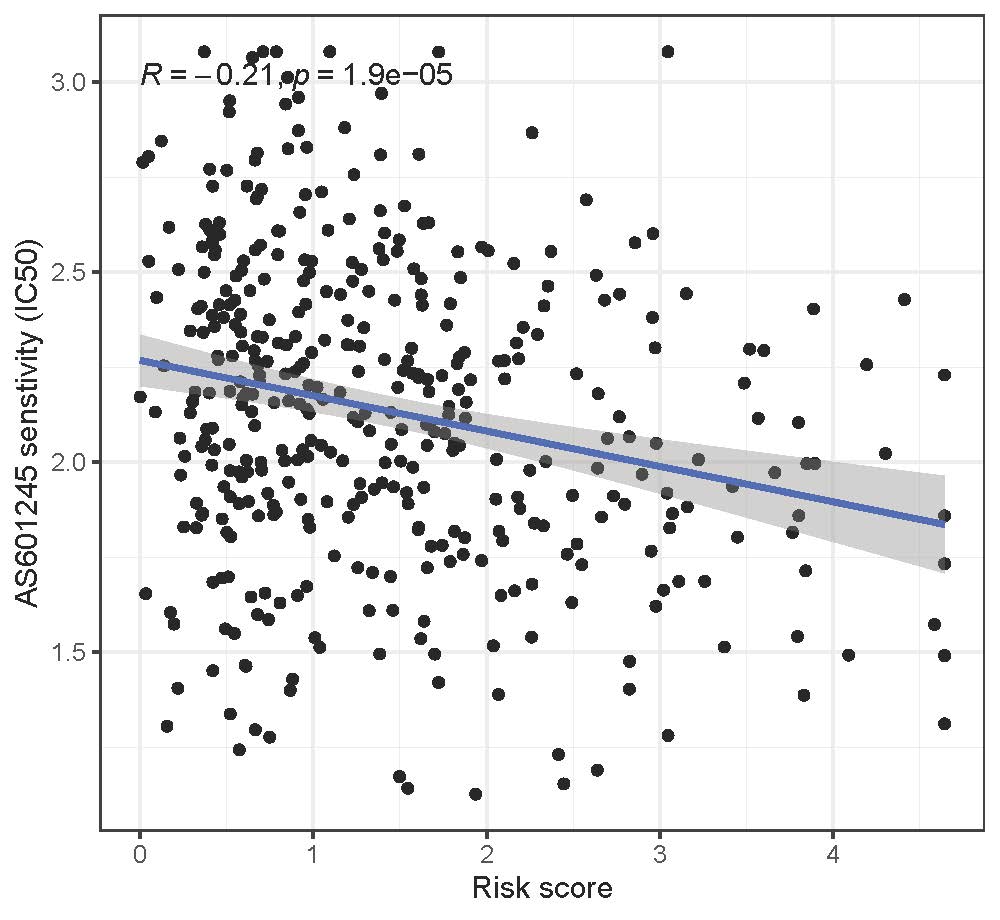

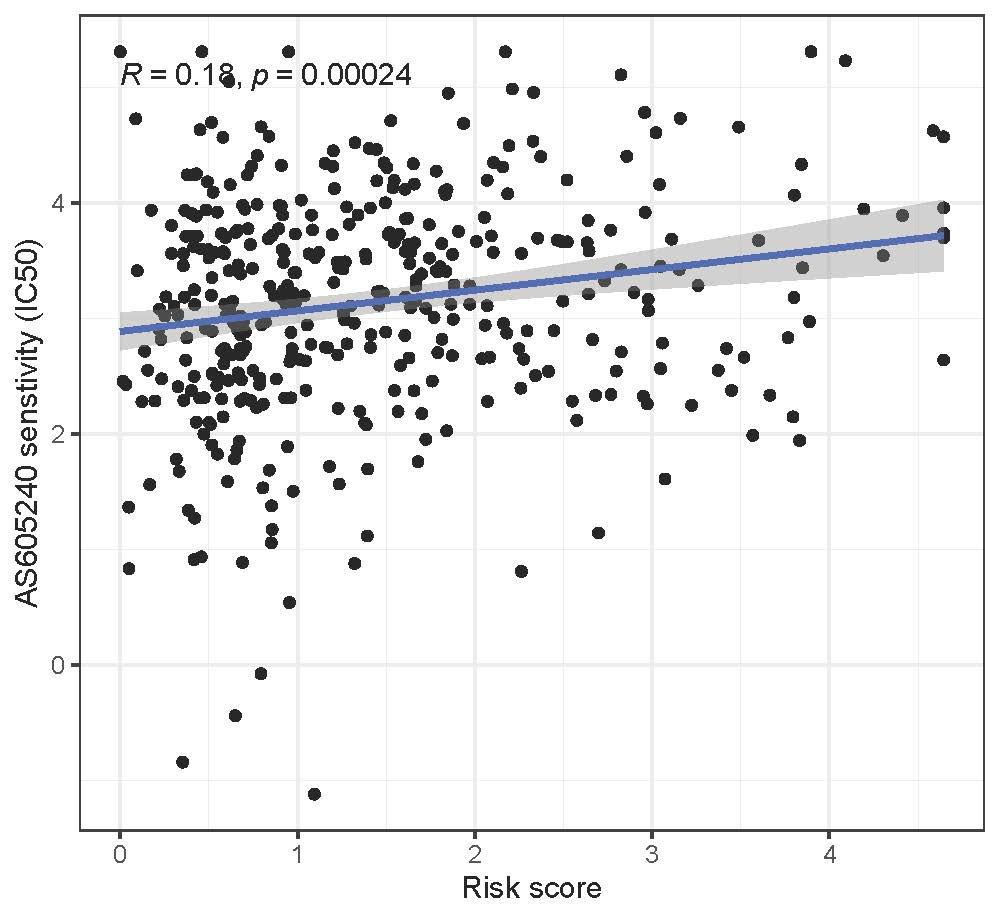

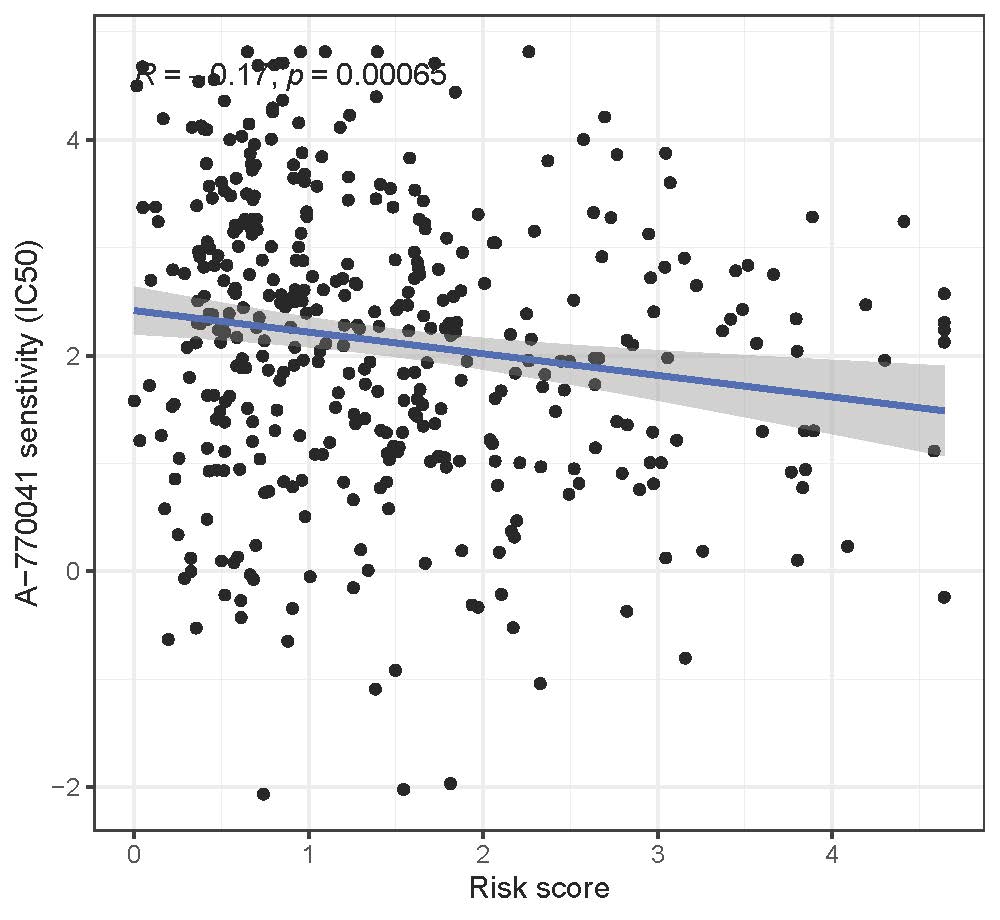

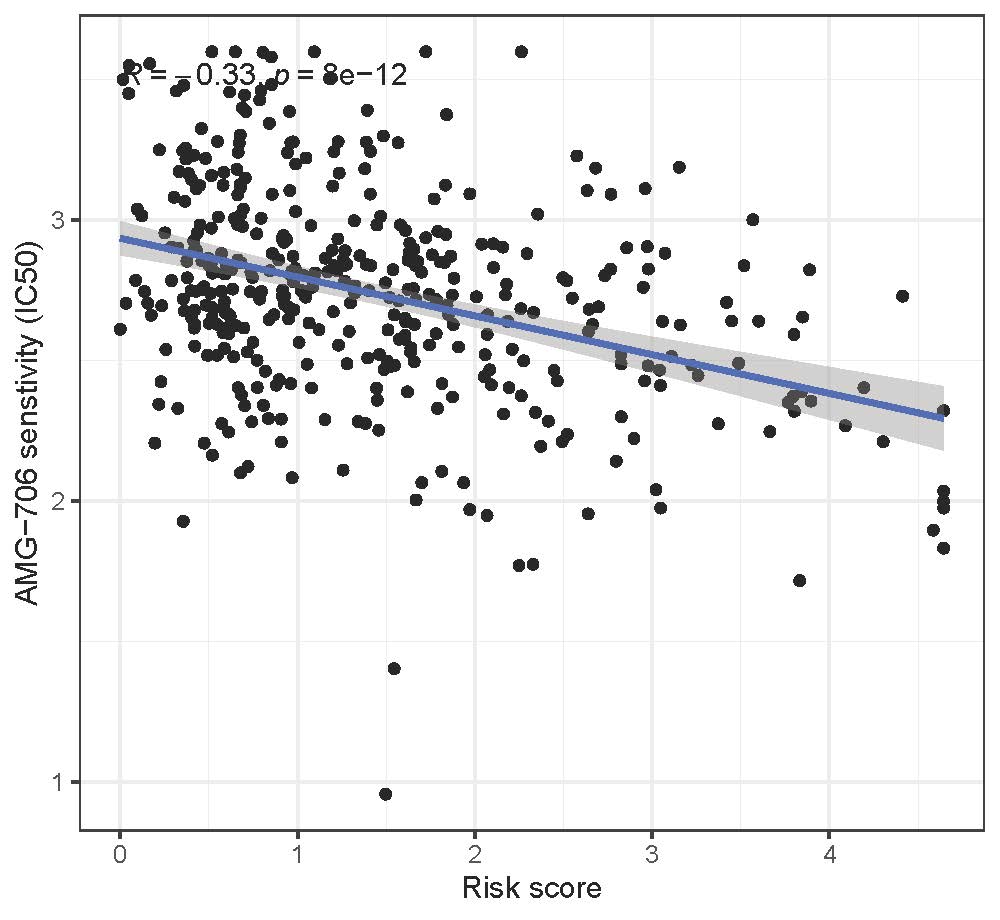


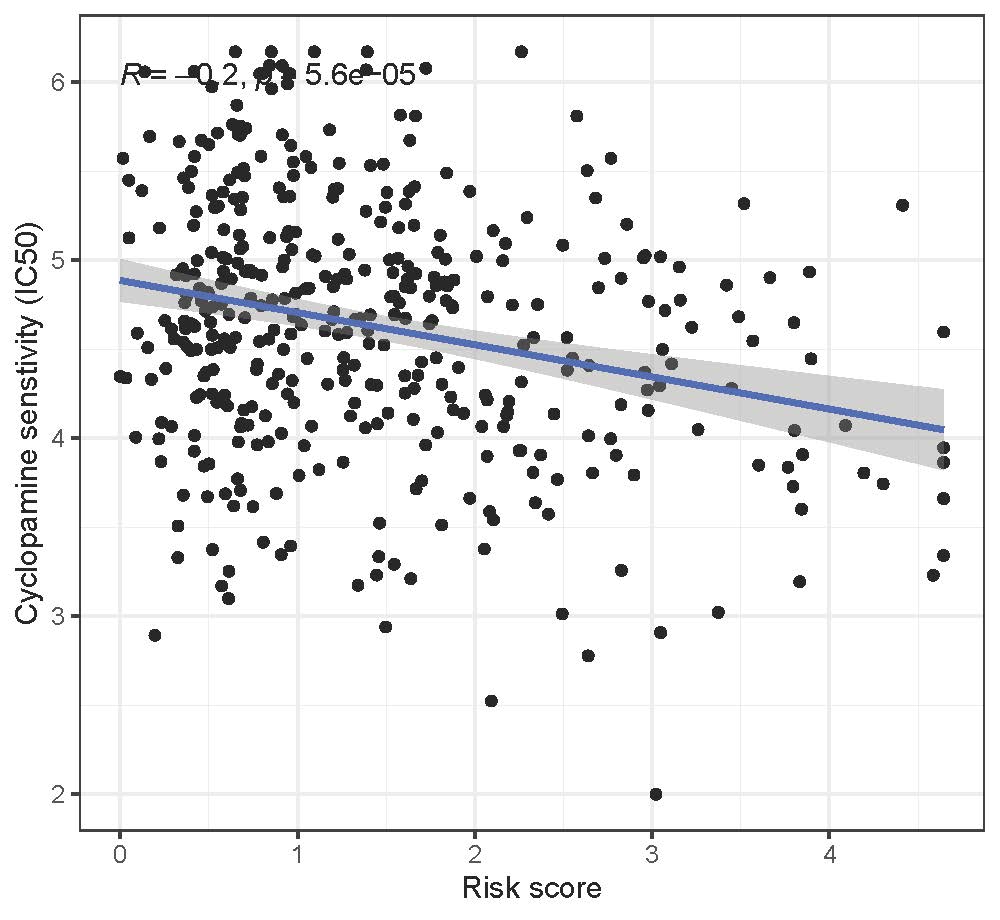

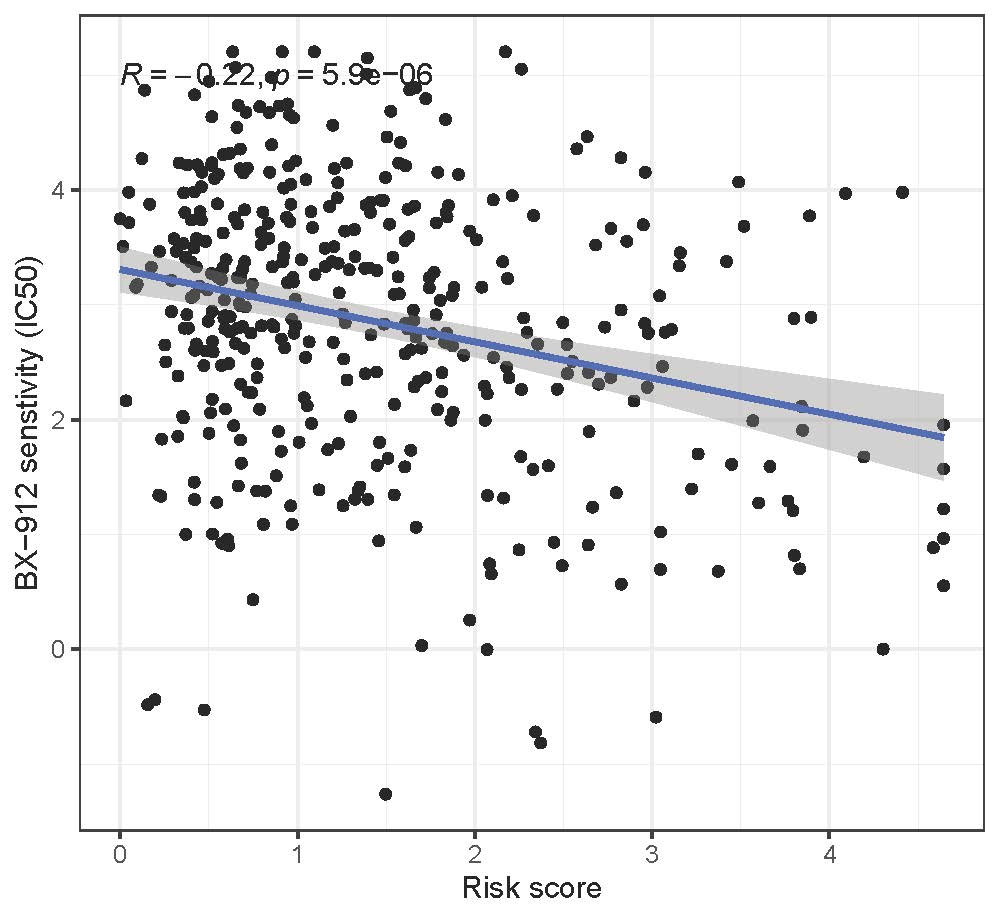

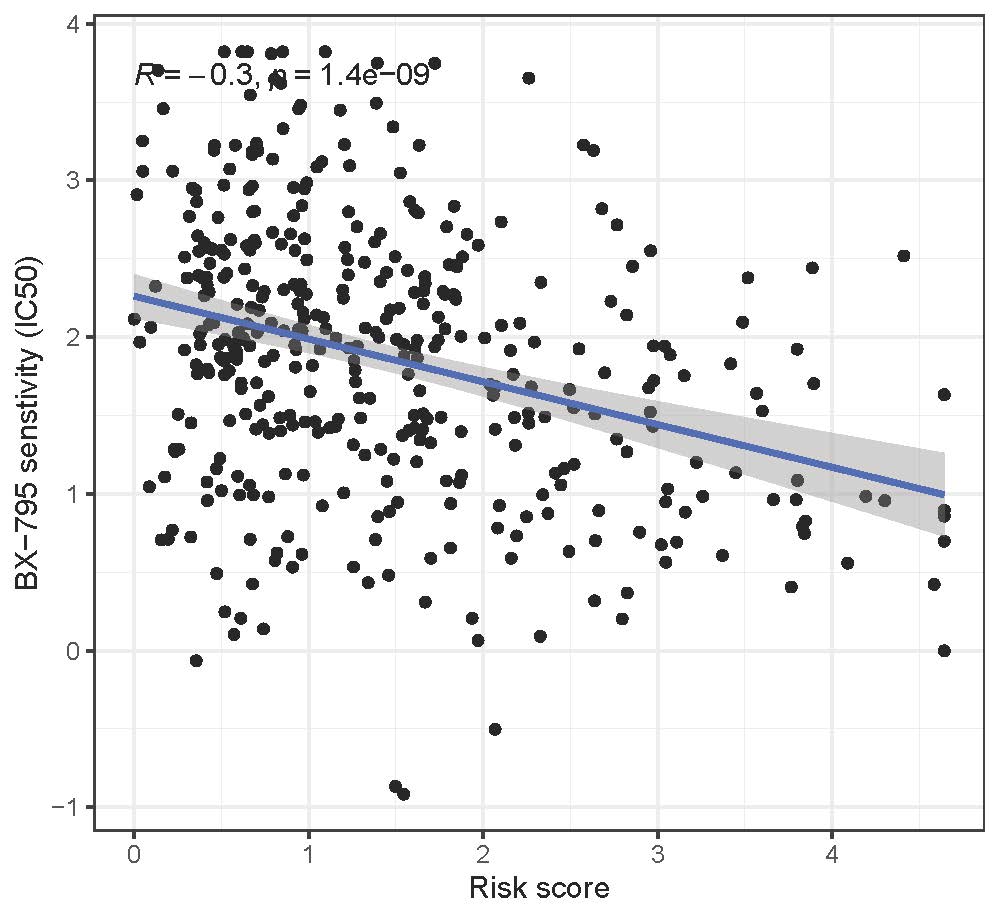

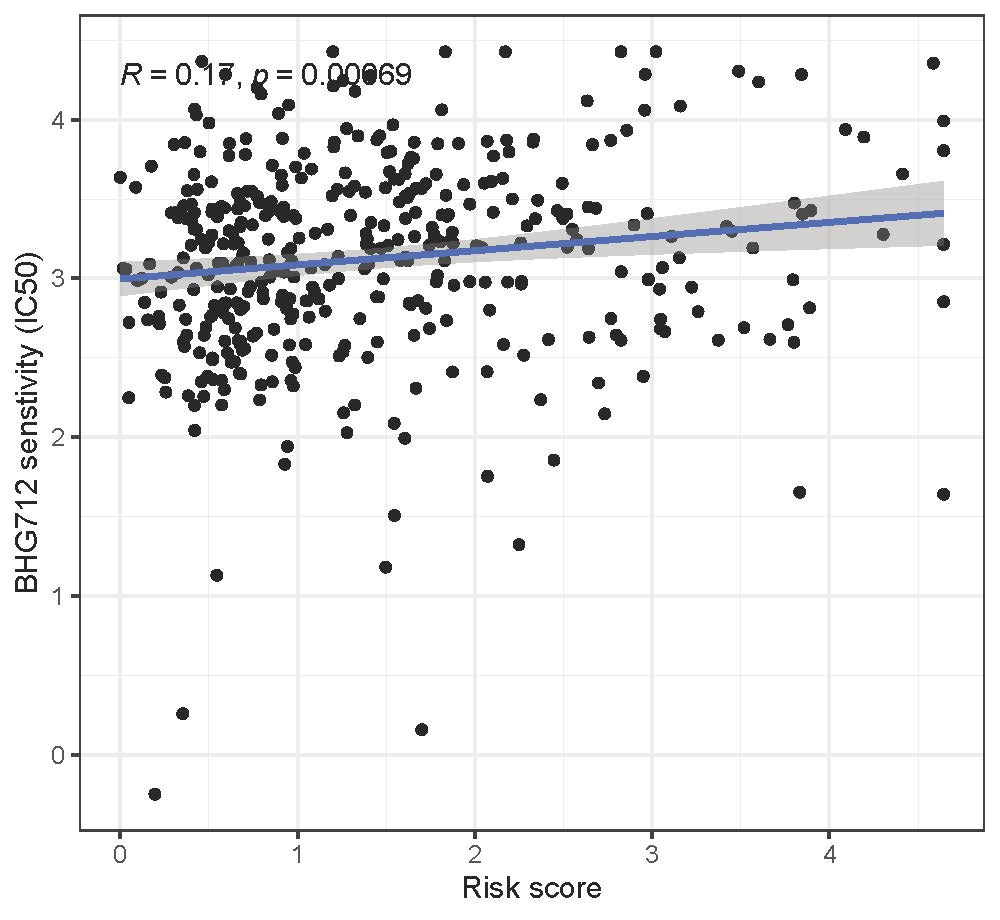


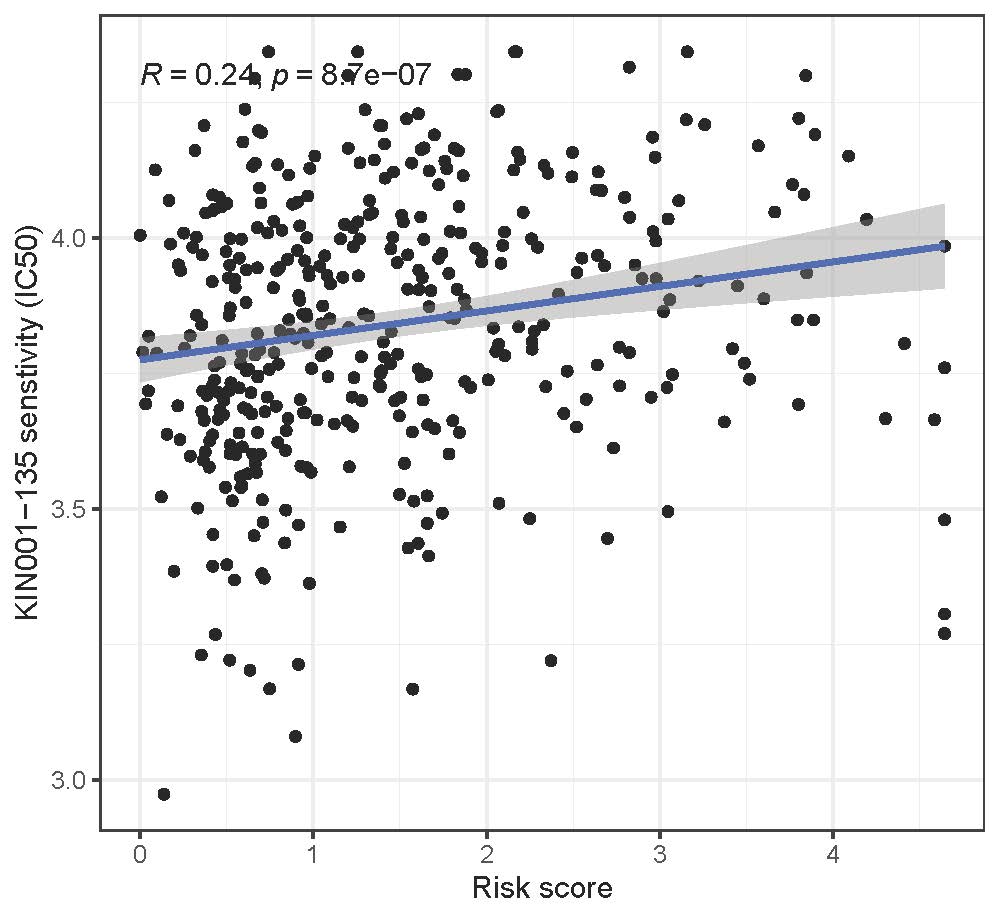

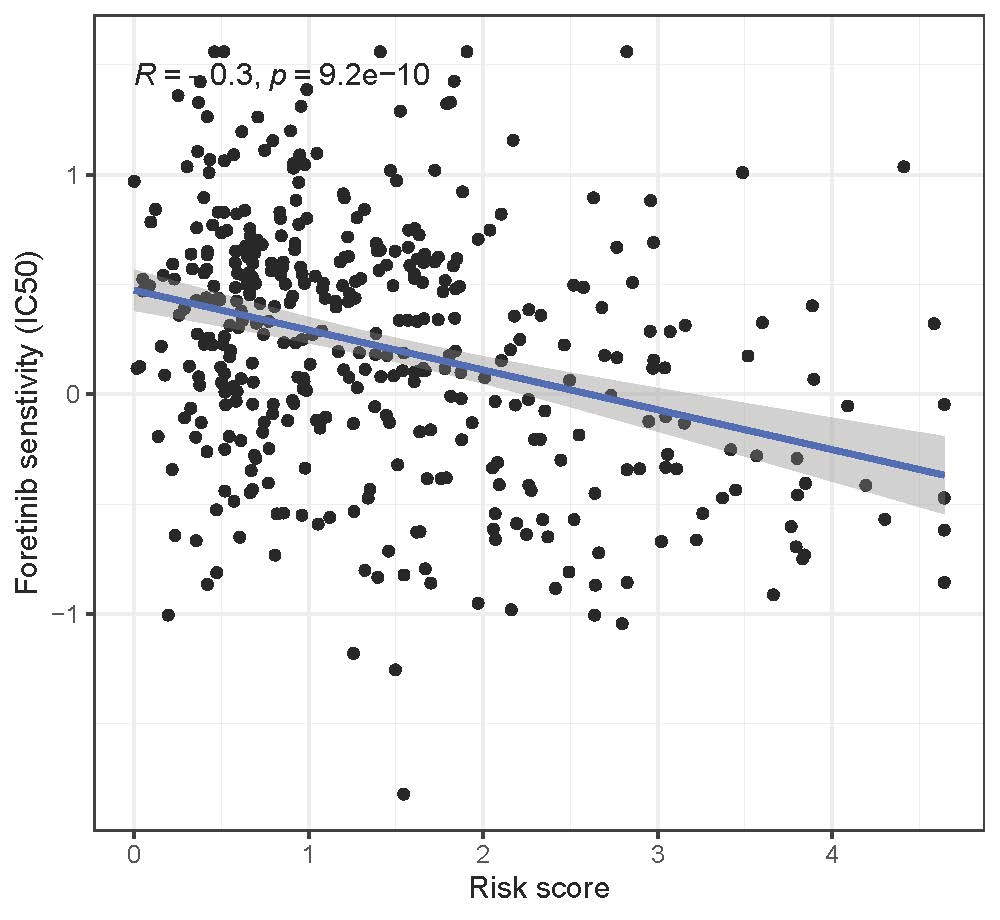

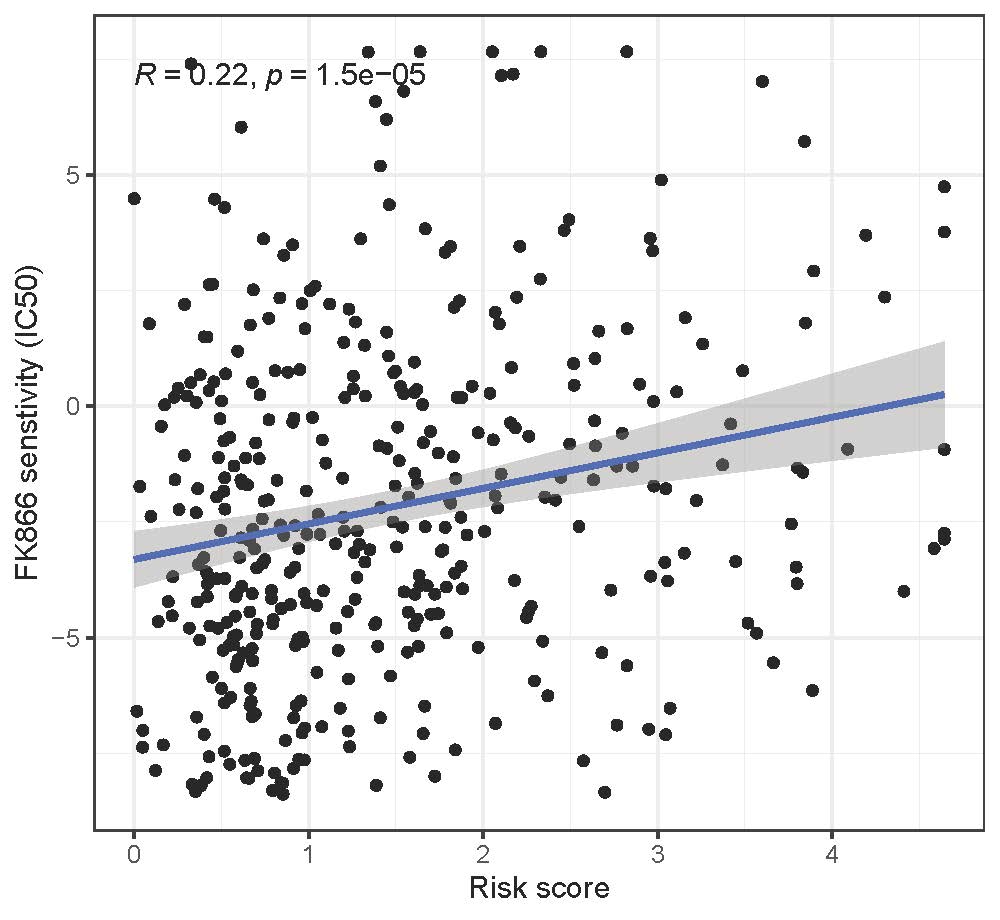

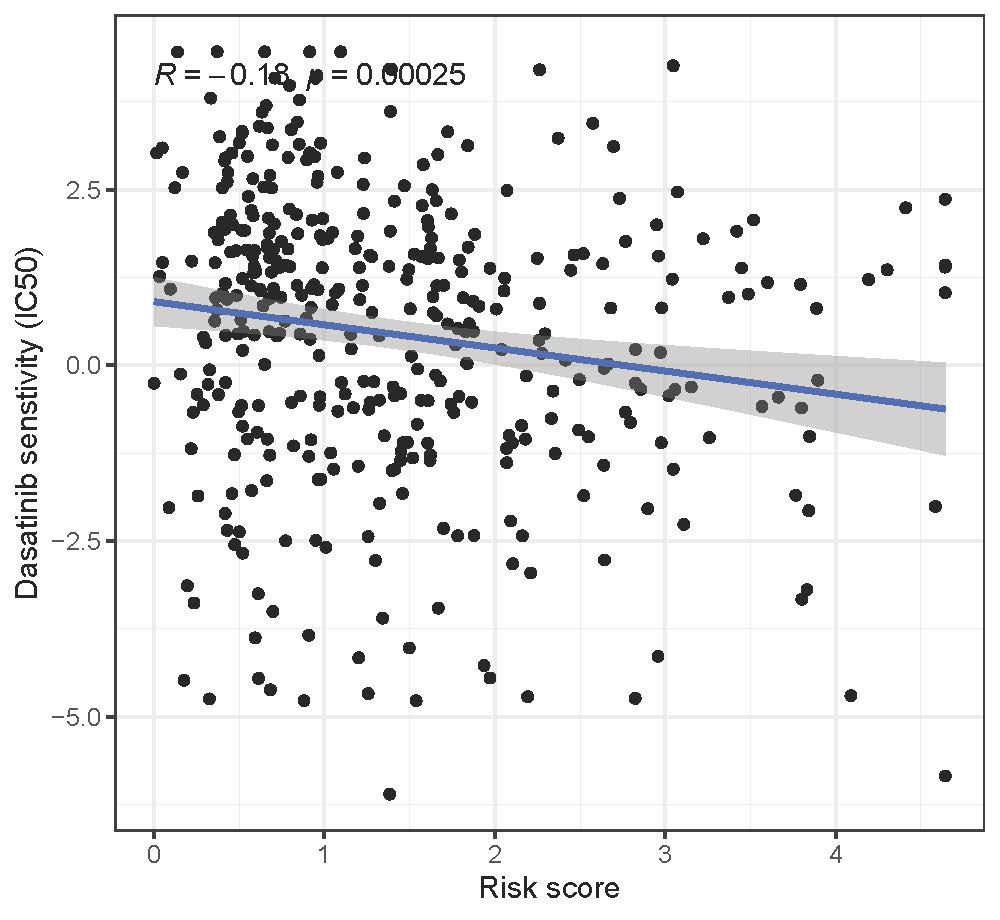


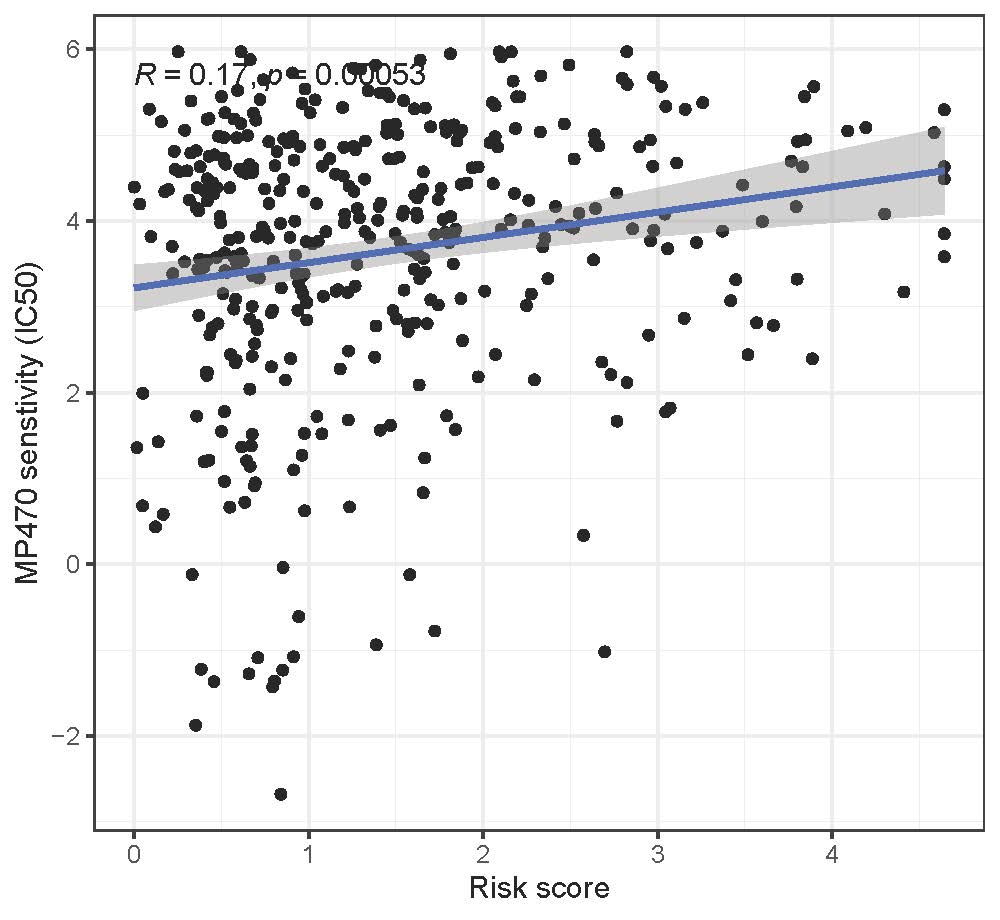

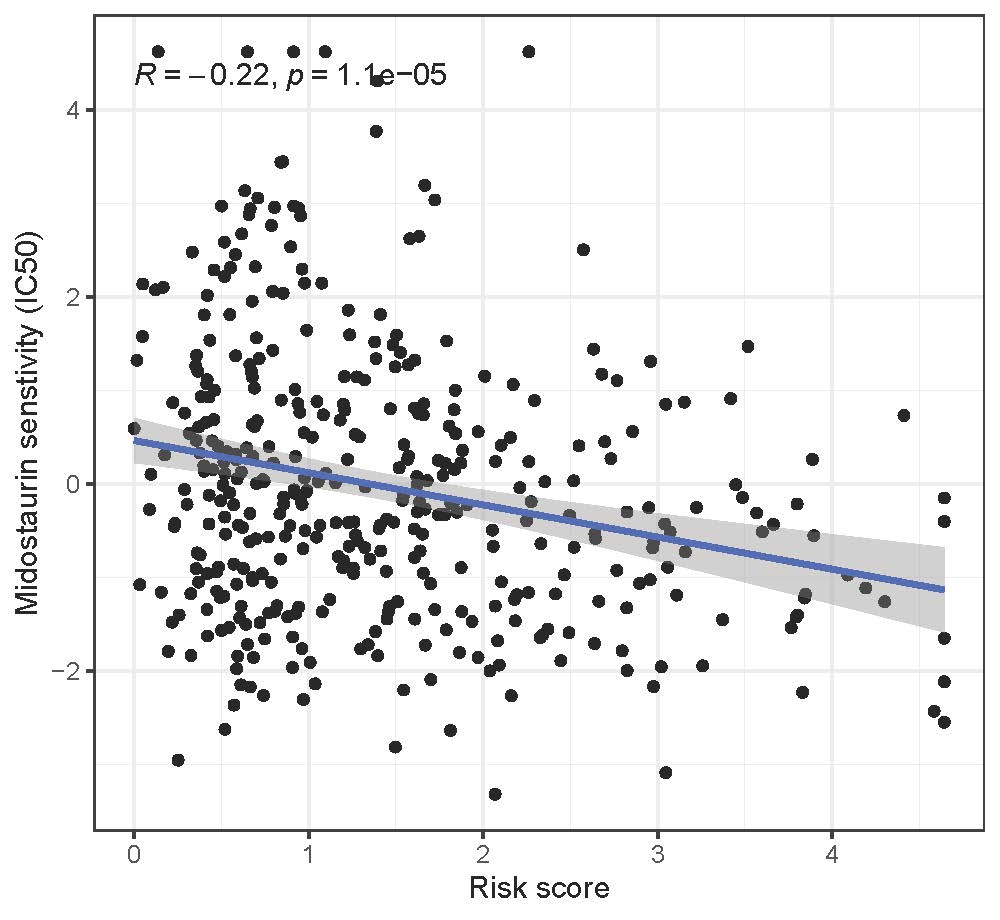

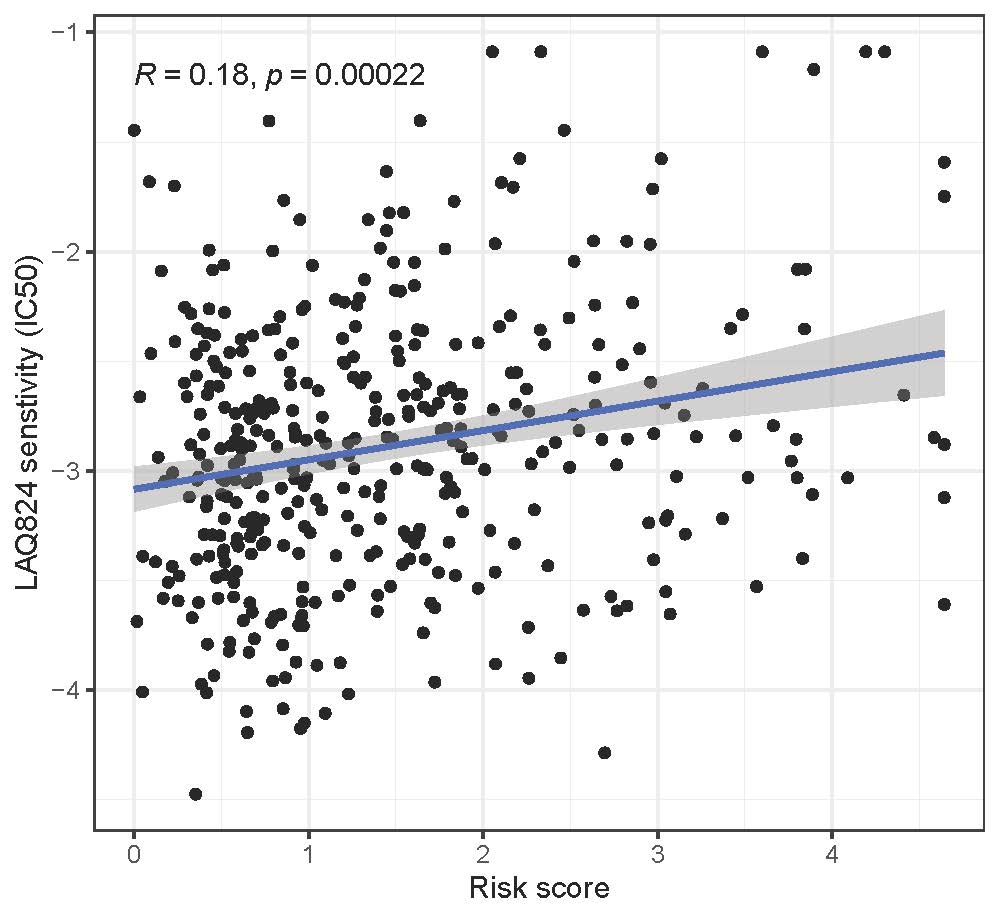

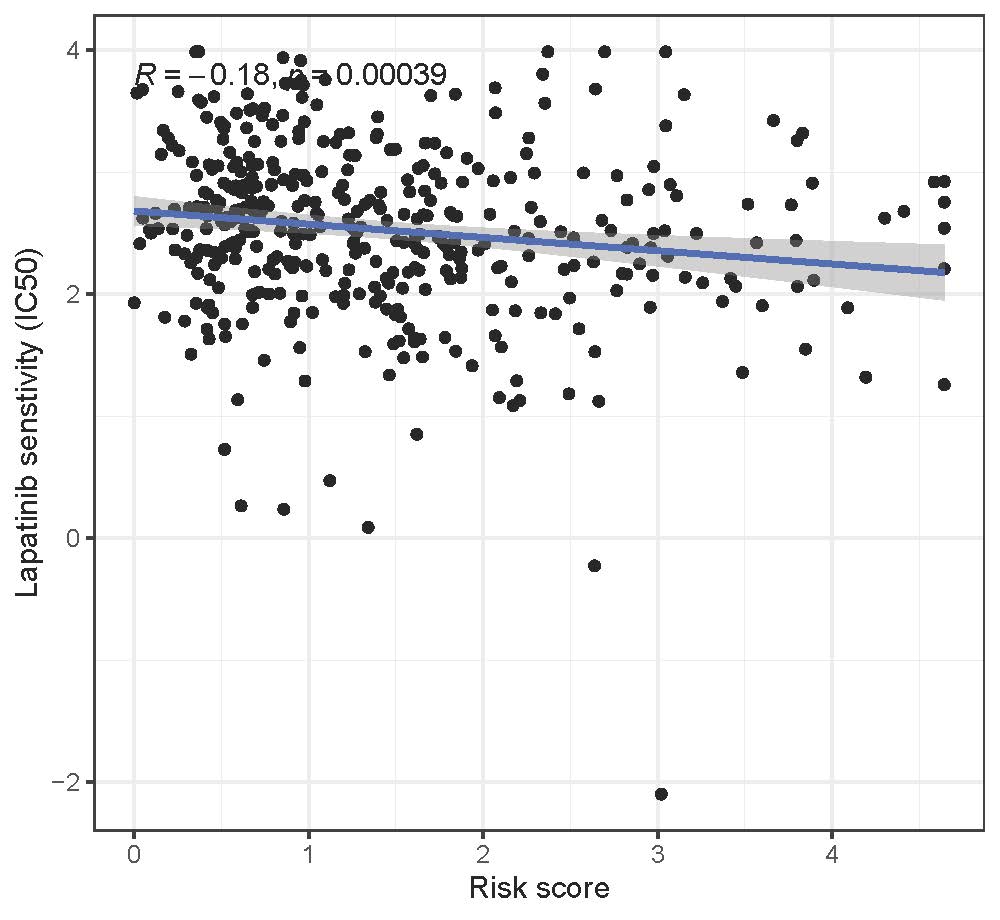


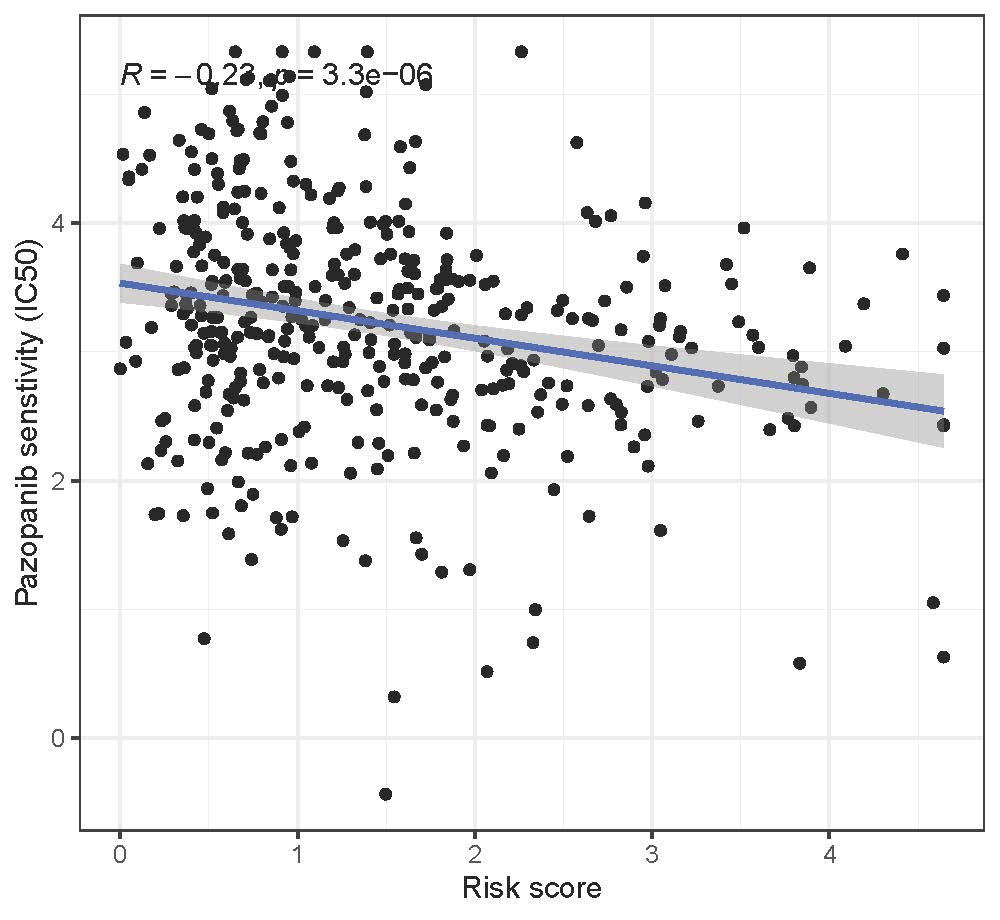


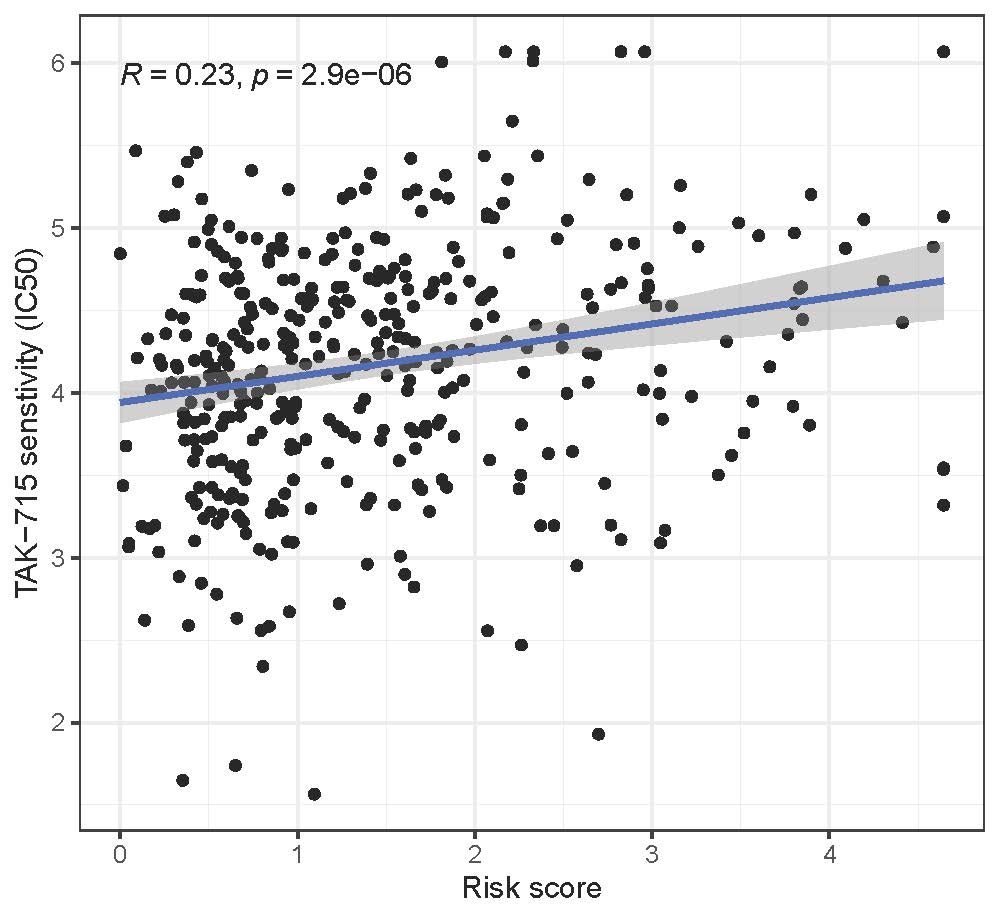

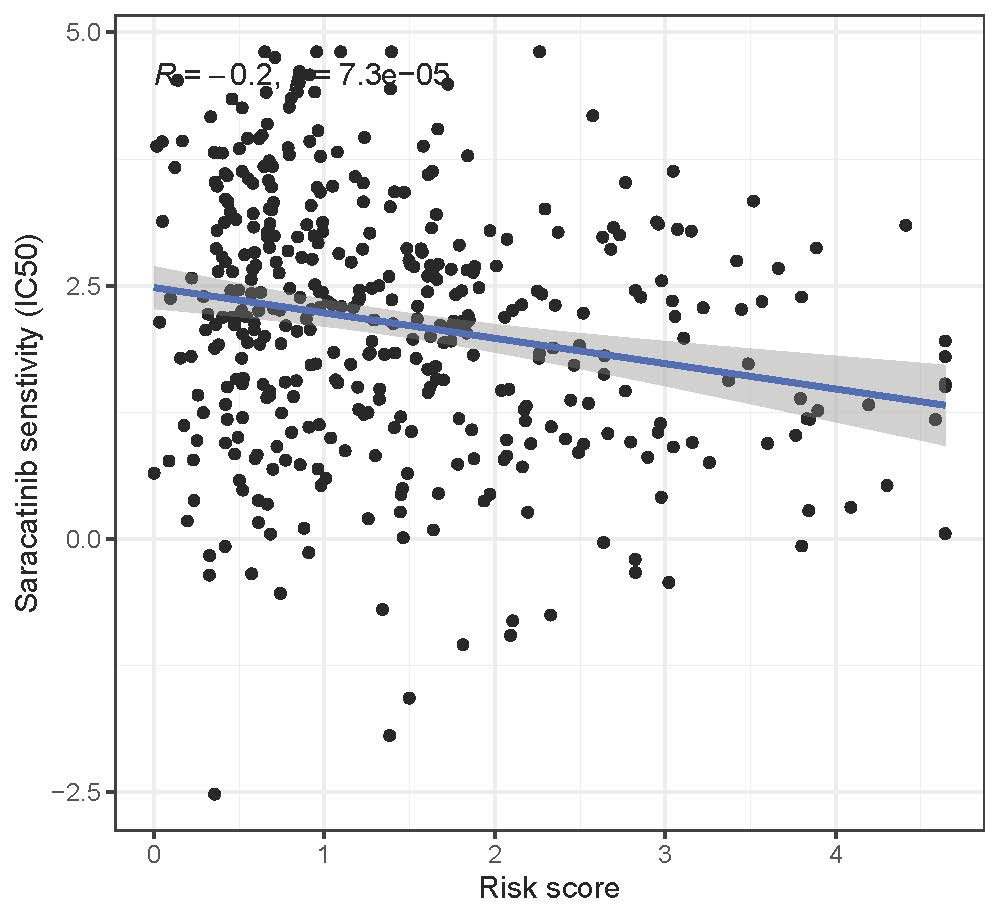

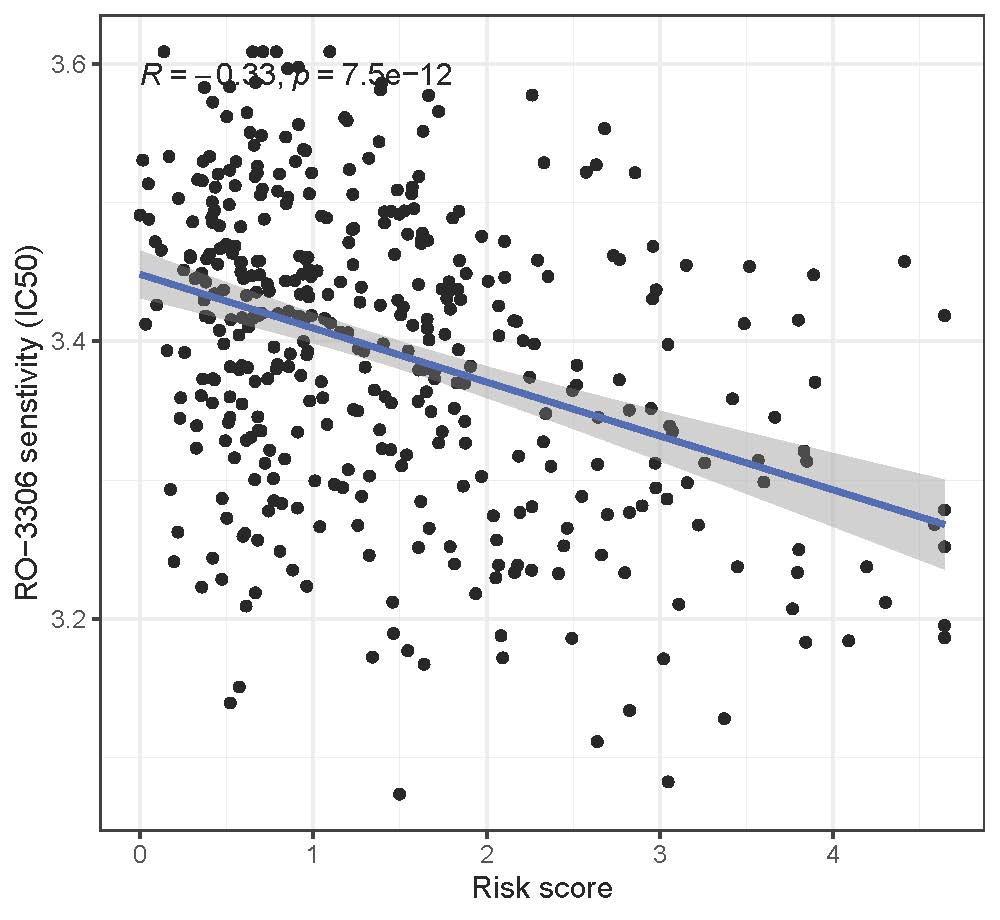


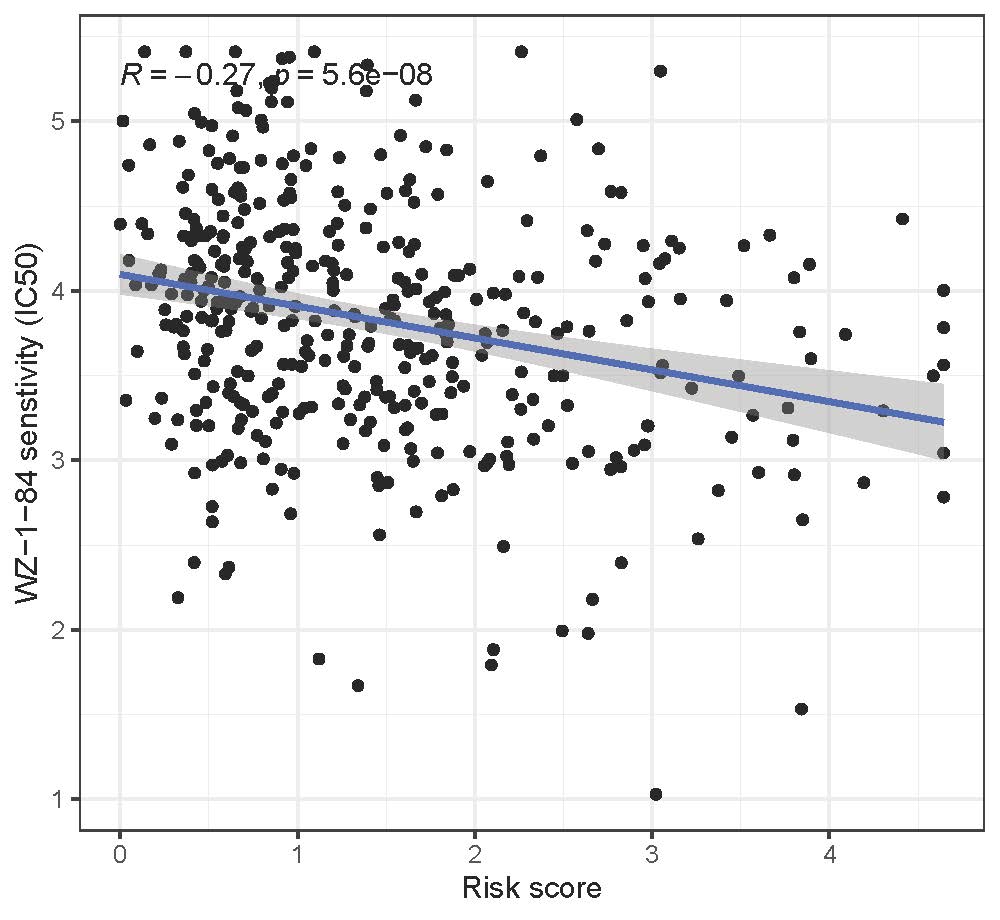

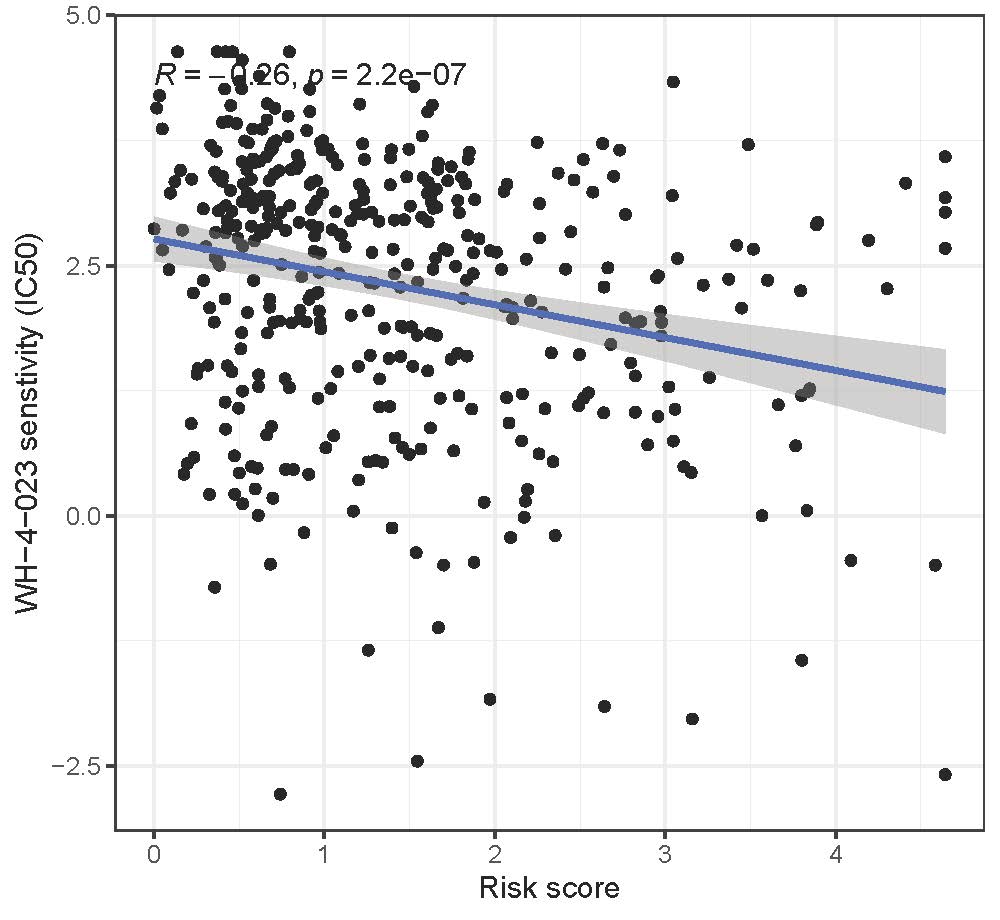

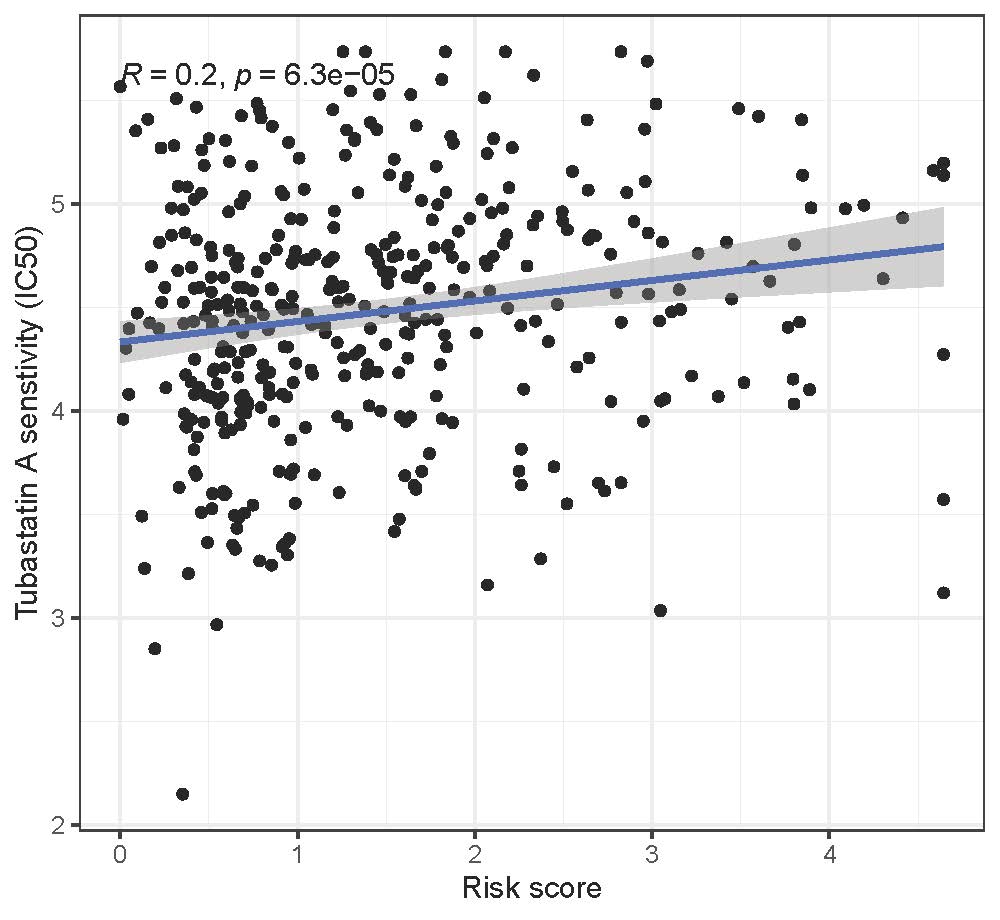

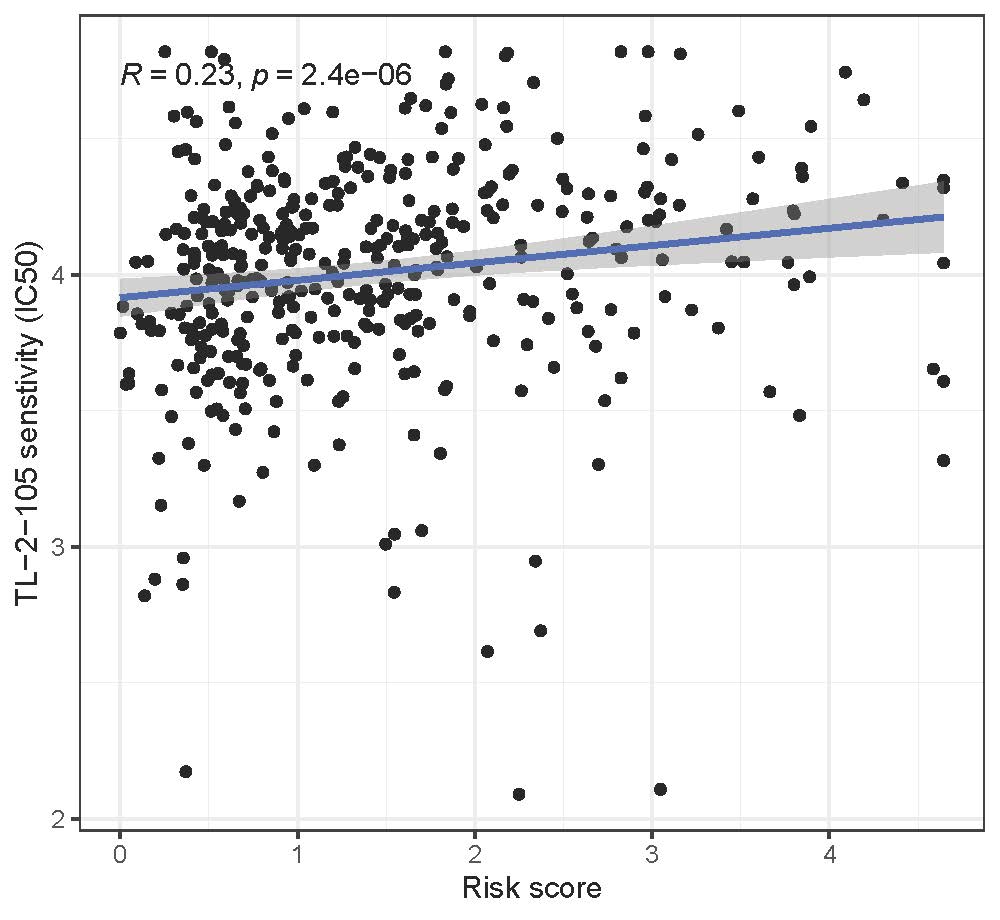


Supplementary Figure S1: Scatter plot between risk score and drug sensitivity.

Supplement: Supplementary file 1 — Additional File Figure S1: Scatter plot between risk score and drug sensitivity. [file 12894_2023_1292_MOESM1_ESM.docx]
