# Supplementary material for: Comprehensive analysis of cuproptosis-related lncRNAs signature to predict prognosis in bladder urothelial carcinoma
Source: BMC Urol. 2023 Jul 21;23:124. doi: 10.1186/s12894-023-01292-9 (PMC10362680; doi:10.1186/s12894-023-01292-9)
Supplement: Supplementary file 2 — Additional File Table S1: 19 cuproptosis-related genes. [file 12894_2023_1292_MOESM2_ESM.docx]

Supplementary Table S1: 19 cuproptosis-related genes.
