# Supplementary material for: Comprehensive analysis of cuproptosis-related lncRNAs signature to predict prognosis in bladder urothelial carcinoma
Source: BMC Urol. 2023 Jul 21;23:124. doi: 10.1186/s12894-023-01292-9 (PMC10362680; doi:10.1186/s12894-023-01292-9)
Supplement: Supplementary file 3 — Additional File Table S2: Data of differentially expressed genes. [file 12894_2023_1292_MOESM3_ESM.docx]

Supplementary Table S2: Data of differentially expressed genes.

| gene | lowMean | highMean | logFC | pValue | fdr |
| --- | --- | --- | --- | --- | --- |
| RPL37P1 | 5.31403 | 2.420621 | -1.13443 | 1.80E-12 | 1.39E-10 |
| BTNL9 | 4.889147 | 2.392049 | -1.03134 | 5.84E-05 | 0.000226 |
| SLC39A14 | 13.27673 | 27.45747 | 1.048299 | 1.20E-17 | 2.15E-14 |
| ANKRD1 | 1.053013 | 4.040007 | 1.939835 | 0.001183 | 0.003042 |
| AC027020.2 | 3.007951 | 1.433333 | -1.06941 | 3.34E-13 | 4.00E-11 |
| BHMT | 53.98604 | 25.15381 | -1.10181 | 3.82E-07 | 3.22E-06 |
| KPNA7 | 1.11175 | 2.22595 | 1.001589 | 1.46E-06 | 9.94E-06 |
| PTPRD | 0.766212 | 1.726922 | 1.172388 | 9.11E-06 | 4.65E-05 |
| AQP7 | 3.189257 | 1.504668 | -1.08377 | 0.000491 | 0.001412 |
| CXCL6 | 8.94878 | 19.87981 | 1.151541 | 9.25E-05 | 0.000334 |
| B3GAT1 | 2.076438 | 0.982306 | -1.07987 | 2.52E-06 | 1.56E-05 |
| LINC01451 | 6.691226 | 3.180889 | -1.07284 | 0.000253 | 0.000794 |
| KRT6B | 80.75913 | 422.2368 | 2.386355 | 8.48E-05 | 0.000309 |
| AC079466.1 | 8.803159 | 0.414496 | -4.40859 | 0.000246 | 0.000777 |
| PRSS3P4 | 2.666554 | 0.964031 | -1.46783 | 0.002023 | 0.004861 |
| SUGCT | 2.71249 | 8.621183 | 1.668268 | 1.56E-09 | 3.25E-08 |
| AP005432.2 | 71.37312 | 20.69526 | -1.78608 | 0.0003 | 0.00092 |
| SYNM | 13.36886 | 28.42326 | 1.088195 | 1.01E-05 | 5.10E-05 |
| PPP1R14C | 13.8137 | 32.85199 | 1.249881 | 8.59E-06 | 4.42E-05 |
| CACNA2D1 | 2.130648 | 4.821699 | 1.178249 | 3.80E-07 | 3.21E-06 |
| RHEX | 9.057543 | 3.958237 | -1.19426 | 0.006868 | 0.014009 |
| MYOCD | 0.943176 | 2.267476 | 1.265489 | 0.001011 | 0.002651 |
| LINC01614 | 3.984837 | 10.27776 | 1.366934 | 1.51E-08 | 2.17E-07 |
| AL355916.1 | 1.022752 | 2.38734 | 1.222947 | 8.38E-06 | 4.33E-05 |
| AC105460.1 | 5.779422 | 12.93379 | 1.162148 | 0.003771 | 0.008293 |
| RNF224 | 2.1243 | 0.976602 | -1.12114 | 0.000509 | 0.001454 |
| VSNL1 | 5.503184 | 12.96987 | 1.236826 | 1.47E-06 | 1.00E-05 |
| C2orf66 | 2.568294 | 1.173112 | -1.13047 | 2.64E-07 | 2.37E-06 |
| CSDC2 | 2.895366 | 6.06979 | 1.067901 | 2.86E-07 | 2.52E-06 |
| TGFBI | 78.18661 | 224.3348 | 1.52066 | 2.02E-12 | 1.52E-10 |
| JAM3 | 2.025227 | 5.096265 | 1.331357 | 2.79E-09 | 5.30E-08 |
| SH3GL3 | 0.573525 | 1.520785 | 1.406888 | 7.15E-05 | 0.000268 |
| ITGA2 | 21.42582 | 46.43084 | 1.115733 | 8.09E-11 | 3.01E-09 |
| AC243960.1 | 1.978714 | 0.981247 | -1.01188 | 0.027777 | 0.046905 |
| AC008739.2 | 3.898819 | 1.331257 | -1.55025 | 1.33E-12 | 1.13E-10 |
| RNU6-403P | 2.351614 | 6.142134 | 1.385089 | 2.55E-05 | 0.000111 |
| PRSS12 | 1.713145 | 3.986358 | 1.218424 | 8.52E-06 | 4.39E-05 |
| SPRR2E | 10.79968 | 111.7948 | 3.371792 | 9.45E-06 | 4.80E-05 |
| TNNI2 | 154.1134 | 76.62379 | -1.00813 | 0.000352 | 0.001055 |
| SHD | 1.68334 | 0.815468 | -1.04563 | 1.62E-06 | 1.09E-05 |
| FGF19 | 3.502964 | 10.08746 | 1.525915 | 0.007289 | 0.01477 |
| RAET1L | 2.285039 | 5.771255 | 1.336666 | 5.10E-07 | 4.12E-06 |
| LAMA2 | 2.61351 | 5.260356 | 1.009172 | 4.63E-10 | 1.22E-08 |
| LRRC38 | 0.812316 | 3.391369 | 2.061754 | 0.014567 | 0.026868 |
| AC010201.1 | 1.517439 | 0.74679 | -1.02286 | 1.46E-10 | 4.72E-09 |
| PPBP | 1.553261 | 3.824683 | 1.30004 | 0.000165 | 0.000551 |
| MIR2117HG | 0.850407 | 1.735443 | 1.029079 | 4.49E-05 | 0.00018 |
| SLC7A11 | 4.807007 | 17.18721 | 1.838125 | 2.38E-05 | 0.000105 |
| RPL10P13 | 0.791601 | 1.610112 | 1.024316 | 0.000446 | 0.001296 |
| OLFML2A | 14.41892 | 29.2321 | 1.019591 | 3.51E-15 | 1.09E-12 |
| AC007993.3 | 1.487371 | 0.56528 | -1.39573 | 1.18E-10 | 4.00E-09 |
| MSLN | 16.10873 | 43.73857 | 1.441063 | 0.000382 | 0.001133 |
| TAC3 | 170.5639 | 64.52629 | -1.40235 | 0.00736 | 0.014897 |
| APCDD1L | 1.999168 | 7.880104 | 1.978815 | 8.45E-12 | 4.85E-10 |
| HKDC1 | 0.777399 | 3.855474 | 2.310181 | 0.000103 | 0.000367 |
| MIR3142HG | 2.239433 | 1.101298 | -1.02393 | 0.001924 | 0.004648 |
| LINC00930 | 7.821862 | 2.285497 | -1.775 | 7.54E-10 | 1.78E-08 |
| PLN | 5.997906 | 15.25807 | 1.347041 | 1.93E-05 | 8.78E-05 |
| CDH11 | 6.428092 | 13.03254 | 1.019655 | 1.62E-07 | 1.56E-06 |
| AMY2B | 1.993258 | 0.994937 | -1.00245 | 7.61E-10 | 1.79E-08 |
| AC010719.1 | 4.550291 | 2.145448 | -1.08468 | 9.98E-10 | 2.24E-08 |
| RASL10A | 4.251547 | 2.048945 | -1.05311 | 5.48E-07 | 4.37E-06 |
| KRT20 | 393.3519 | 184.1709 | -1.09478 | 0.000141 | 0.000482 |
| NXPH3 | 1.62547 | 3.251513 | 1.000254 | 0.000424 | 0.001238 |
| KRT6C | 10.98343 | 102.4534 | 3.221568 | 9.44E-05 | 0.000339 |
| AC005224.3 | 0.661032 | 1.608901 | 1.283284 | 0.010688 | 0.020569 |
| AHNAK2 | 5.522977 | 21.02141 | 1.928341 | 3.78E-14 | 7.64E-12 |
| PGF | 9.419136 | 19.59754 | 1.057006 | 9.17E-07 | 6.69E-06 |
| CDH13 | 1.555473 | 3.170448 | 1.027333 | 3.64E-08 | 4.47E-07 |
| ABCA4 | 1.399687 | 6.190972 | 2.145061 | 2.65E-06 | 1.63E-05 |
| SYNPO2 | 6.649662 | 16.37571 | 1.300204 | 9.42E-05 | 0.000339 |
| NID2 | 3.940579 | 10.01736 | 1.346023 | 8.30E-13 | 7.81E-11 |
| CES1 | 32.57894 | 165.9264 | 2.348532 | 1.65E-06 | 1.10E-05 |
| DSC2 | 12.8837 | 35.17148 | 1.44886 | 2.36E-07 | 2.15E-06 |
| ATP6V0A4 | 1.975958 | 5.347897 | 1.436419 | 0.00264 | 0.00611 |
| B3GAT1-DT | 2.995239 | 1.044149 | -1.52034 | 9.00E-08 | 9.54E-07 |
| MUC4 | 5.418245 | 13.13499 | 1.277518 | 0.01976 | 0.034904 |
| MMP9 | 43.25601 | 167.1954 | 1.950563 | 0.00244 | 0.005709 |
| MIR7152 | 5.373031 | 2.489649 | -1.10979 | 1.07E-05 | 5.36E-05 |
| CCN3 | 3.737699 | 8.425366 | 1.172589 | 3.29E-05 | 0.000138 |
| CCDC198 | 3.631747 | 1.570692 | -1.20926 | 8.25E-06 | 4.27E-05 |
| SHROOM1 | 47.6989 | 21.29781 | -1.16325 | 1.76E-08 | 2.45E-07 |
| KRT5 | 1020.099 | 2810.527 | 1.462132 | 0.000232 | 0.000735 |
| RAB23 | 4.430194 | 8.952082 | 1.014853 | 1.28E-14 | 3.14E-12 |
| TUBAL3 | 0.366204 | 1.828103 | 2.319628 | 4.31E-06 | 2.48E-05 |
| SPRR2G | 3.635042 | 49.09406 | 3.755505 | 0.007952 | 0.015936 |
| SPRR2A | 36.68044 | 185.4241 | 2.337746 | 0.000168 | 0.000559 |
| CYP26B1 | 3.304666 | 8.919815 | 1.432509 | 5.31E-06 | 2.96E-05 |
| AC018978.1 | 0.762561 | 2.933918 | 1.943903 | 4.28E-10 | 1.15E-08 |
| FGF7 | 3.167593 | 7.418803 | 1.2278 | 7.79E-08 | 8.44E-07 |
| ITPKA | 2.600655 | 5.403817 | 1.055104 | 0.015219 | 0.027942 |
| AP000424.2 | 1.697888 | 0.658841 | -1.36574 | 0.00212 | 0.005056 |
| TBX1 | 35.20366 | 15.99798 | -1.13784 | 1.69E-05 | 7.84E-05 |
| CASP14 | 83.22324 | 174.9761 | 1.0721 | 0.021263 | 0.037153 |
| FBN2 | 5.7141 | 12.90122 | 1.174909 | 5.86E-06 | 3.21E-05 |
| KRT4 | 83.94643 | 195.9161 | 1.222695 | 0.001404 | 0.003529 |
| AC005009.1 | 2.165339 | 0.716778 | -1.595 | 7.38E-10 | 1.75E-08 |
| AC073534.2 | 2.693962 | 0.931764 | -1.53169 | 1.14E-21 | 9.22E-18 |
| SLC2A3 | 16.60868 | 35.09823 | 1.079461 | 1.92E-07 | 1.81E-06 |
| PI3 | 593.7453 | 2455.723 | 2.048232 | 0.001966 | 0.004737 |
| SPRR4 | 0.779527 | 4.444344 | 2.5113 | 0.006063 | 0.012587 |
| UNC5C | 0.673141 | 1.503291 | 1.159144 | 5.75E-07 | 4.54E-06 |
| LAYN | 5.227332 | 10.94215 | 1.06575 | 1.76E-07 | 1.68E-06 |
| AC004080.1 | 13.74155 | 4.506603 | -1.60843 | 0.000109 | 0.000385 |
| ATP6V0CP2 | 4.906885 | 1.890461 | -1.37607 | 1.30E-06 | 8.97E-06 |
| TENT5B | 7.958767 | 16.3548 | 1.039097 | 5.88E-08 | 6.70E-07 |
| TTC6 | 1.5707 | 0.715052 | -1.13529 | 3.14E-09 | 5.89E-08 |
| EVA1A | 4.618707 | 9.716603 | 1.072963 | 8.05E-07 | 6.01E-06 |
| TGFB2 | 1.817646 | 4.525878 | 1.316127 | 3.74E-10 | 1.03E-08 |
| CCL24 | 2.790324 | 6.647228 | 1.25232 | 0.000262 | 0.000817 |
| CCN4 | 7.486598 | 15.01723 | 1.004236 | 1.15E-07 | 1.18E-06 |
| MIR200B | 5.855996 | 2.755482 | -1.08761 | 1.64E-05 | 7.64E-05 |
| PF4V1 | 0.680504 | 1.444613 | 1.086006 | 2.68E-05 | 0.000116 |
| HMCN1 | 0.73324 | 2.363374 | 1.688491 | 6.81E-08 | 7.53E-07 |
| SPRR2D | 18.67763 | 92.36454 | 2.306028 | 0.000125 | 0.000432 |
| PCSK1 | 0.728649 | 2.351314 | 1.690171 | 3.27E-07 | 2.83E-06 |
| CYP4F23P | 25.2461 | 11.47732 | -1.13728 | 1.35E-09 | 2.87E-08 |
| MAOB | 8.007834 | 17.59559 | 1.13573 | 1.38E-05 | 6.60E-05 |
| AIFM3 | 9.2694 | 3.025466 | -1.61532 | 2.37E-07 | 2.16E-06 |
| BEX1 | 2.512106 | 13.3946 | 2.414682 | 0.011596 | 0.022098 |
| AL354919.2 | 2.157475 | 0.783697 | -1.46098 | 2.26E-05 | 0.0001 |
| AHNAK | 83.20615 | 169.7781 | 1.028888 | 2.25E-19 | 6.05E-16 |
| FOXC2 | 0.889859 | 3.236755 | 1.862899 | 5.32E-07 | 4.27E-06 |
| GREM1 | 8.926199 | 18.14318 | 1.023309 | 1.10E-08 | 1.69E-07 |
| ZBED2 | 5.876639 | 14.80486 | 1.333008 | 6.25E-05 | 0.000239 |
| GASAL1 | 1.501505 | 3.014973 | 1.005736 | 3.45E-08 | 4.27E-07 |
| KIRREL1 | 8.16503 | 18.67119 | 1.193284 | 7.64E-17 | 7.72E-14 |
| AC010487.1 | 9.459536 | 3.91577 | -1.27247 | 5.85E-12 | 3.60E-10 |
| COL5A2 | 62.97511 | 134.0484 | 1.0899 | 1.29E-12 | 1.10E-10 |
| MIR1270 | 1.702076 | 0.835434 | -1.0267 | 5.79E-06 | 3.18E-05 |
| CCN5 | 3.073092 | 6.590765 | 1.100755 | 0.028359 | 0.047757 |
| ODAPH | 1.222616 | 4.032594 | 1.721737 | 0.025089 | 0.042975 |
| ZNF737 | 18.33399 | 7.988023 | -1.19861 | 6.28E-08 | 7.09E-07 |
| FOXD1 | 1.496547 | 3.073603 | 1.038294 | 4.80E-05 | 0.00019 |
| ATP12A | 1.162219 | 3.642063 | 1.647873 | 0.003308 | 0.007421 |
| ROR1 | 0.848748 | 1.881701 | 1.148629 | 7.29E-08 | 7.99E-07 |
| TENM2 | 1.496955 | 9.412535 | 2.652552 | 3.92E-09 | 7.07E-08 |
| ZFPM2 | 0.948605 | 2.139947 | 1.173695 | 6.36E-10 | 1.57E-08 |
| AC006042.1 | 11.03688 | 5.435173 | -1.02194 | 1.46E-11 | 7.38E-10 |
| CDA | 25.7347 | 60.8132 | 1.240669 | 1.61E-05 | 7.53E-05 |
| AL161669.2 | 5.321335 | 2.528717 | -1.07338 | 0.029132 | 0.048911 |
| FAT2 | 10.37185 | 25.15495 | 1.278169 | 5.39E-05 | 0.00021 |
| ANGPT1 | 1.353 | 2.946851 | 1.123012 | 8.38E-05 | 0.000306 |
| PCSK9 | 2.966089 | 6.627001 | 1.159794 | 2.07E-06 | 1.32E-05 |
| KLK5 | 9.006384 | 73.69901 | 3.032625 | 5.34E-08 | 6.21E-07 |
| HSPB7 | 6.05025 | 15.67424 | 1.373329 | 9.65E-07 | 6.98E-06 |
| KRT34 | 0.951926 | 5.444606 | 2.515906 | 4.09E-07 | 3.41E-06 |
| PTHLH | 13.57109 | 49.66345 | 1.871648 | 2.55E-10 | 7.37E-09 |
| MIR29B2CHG | 2.362404 | 0.9457 | -1.3208 | 1.71E-08 | 2.39E-07 |
| HSPA4L | 2.563938 | 5.849425 | 1.189933 | 2.70E-11 | 1.26E-09 |
| KLK11 | 2.897785 | 12.03201 | 2.053855 | 0.002369 | 0.005568 |
| CYP4F2 | 3.827275 | 0.767836 | -2.31745 | 0.009947 | 0.019325 |
| TRIM31-AS1 | 3.302347 | 1.624145 | -1.02381 | 0.013471 | 0.02514 |
| DENND2C | 2.990582 | 5.998997 | 1.004295 | 4.30E-10 | 1.16E-08 |
| BNC1 | 4.562055 | 13.82847 | 1.599885 | 1.45E-05 | 6.89E-05 |
| ARL14 | 34.50899 | 14.26782 | -1.27421 | 0.001004 | 0.002634 |
| MIR4768 | 17.05292 | 7.547254 | -1.17599 | 0.000336 | 0.001015 |
| CRTAC1 | 69.86298 | 15.13497 | -2.20664 | 0.000491 | 0.001412 |
| SFRP1 | 6.274673 | 15.06788 | 1.263864 | 8.08E-07 | 6.03E-06 |
| AC012354.4 | 2.215311 | 0.778625 | -1.50851 | 2.53E-05 | 0.000111 |
| EFHD1 | 8.327322 | 16.68906 | 1.002978 | 0.006441 | 0.013272 |
| KLK13 | 2.438641 | 7.983808 | 1.711 | 0.001104 | 0.002862 |
| CASC22 | 2.695833 | 0.49812 | -2.43617 | 3.77E-11 | 1.63E-09 |
| IGLON5 | 0.662461 | 2.962973 | 2.161137 | 8.63E-07 | 6.37E-06 |
| CASQ1 | 18.0542 | 8.662188 | -1.05953 | 1.51E-05 | 7.10E-05 |
| CSPG4 | 10.67632 | 24.11425 | 1.175472 | 3.82E-09 | 6.93E-08 |
| SNORD123 | 17.26843 | 8.551954 | -1.01381 | 1.48E-08 | 2.14E-07 |
| LINC00709 | 2.713037 | 0.658945 | -2.04168 | 7.23E-09 | 1.18E-07 |
| MIR6784 | 6.060055 | 2.674441 | -1.18009 | 0.000336 | 0.001015 |
| COL5A1 | 65.19749 | 135.027 | 1.05036 | 1.69E-10 | 5.36E-09 |
| GATA3-AS1 | 20.97036 | 8.849806 | -1.24463 | 4.60E-10 | 1.22E-08 |
| MYH11 | 48.48381 | 105.5538 | 1.122404 | 0.000675 | 0.001859 |
| PLIN5 | 10.10058 | 3.515041 | -1.52283 | 6.05E-15 | 1.69E-12 |
| GPR18 | 1.810532 | 0.781169 | -1.21271 | 0.000326 | 0.000989 |
| S100A7 | 595.185 | 1282.861 | 1.107955 | 0.029388 | 0.049301 |
| SNORD17 | 21.52518 | 49.68517 | 1.20679 | 0.008649 | 0.017132 |
| PCP4 | 23.14522 | 56.11228 | 1.277602 | 0.021783 | 0.037967 |
| FN1 | 271.8738 | 607.9069 | 1.160913 | 5.68E-11 | 2.29E-09 |
| LRTM1 | 2.961122 | 0.260416 | -3.50725 | 1.08E-05 | 5.36E-05 |
| AC099518.2 | 3.628707 | 1.628849 | -1.1556 | 1.35E-07 | 1.35E-06 |
| ENPP1 | 1.077264 | 4.273776 | 1.988139 | 0.000578 | 0.00162 |
| ISLR | 67.23219 | 135.4285 | 1.010307 | 2.39E-06 | 1.49E-05 |
| SEPTIN9-DT | 7.765755 | 3.637074 | -1.09435 | 2.77E-05 | 0.000119 |
| TREM1 | 2.632926 | 8.03422 | 1.609491 | 5.38E-08 | 6.24E-07 |
| AC008870.5 | 1.654043 | 0.794327 | -1.05819 | 0.02219 | 0.038598 |
| DNM3OS | 1.701264 | 3.578584 | 1.072782 | 0.001253 | 0.003196 |
| FMO8P | 3.487121 | 1.253442 | -1.47614 | 2.44E-08 | 3.22E-07 |
| LINC01711 | 1.183974 | 3.094747 | 1.386184 | 6.93E-05 | 0.000261 |
| NMRAL2P | 4.290812 | 8.593629 | 1.002017 | 0.000553 | 0.001561 |
| GPR37 | 1.039952 | 2.560322 | 1.299808 | 4.29E-09 | 7.63E-08 |
| AL137800.1 | 0.722922 | 2.231903 | 1.626361 | 0.020563 | 0.036101 |
| AKR1B15 | 3.653956 | 8.401676 | 1.201218 | 1.90E-05 | 8.66E-05 |
| COL10A1 | 13.58158 | 31.17328 | 1.198659 | 1.37E-08 | 2.02E-07 |
| SULT2B1 | 6.090367 | 13.63639 | 1.162861 | 0.000906 | 0.002409 |
| LINC01269 | 1.184521 | 2.657643 | 1.165843 | 0.000254 | 0.000796 |
| PCDHGC3 | 3.555997 | 9.000882 | 1.339812 | 2.02E-16 | 1.72E-13 |
| AC078880.3 | 4.754304 | 0.851284 | -2.48152 | 6.09E-07 | 4.76E-06 |
| AL691482.3 | 17.94884 | 8.455417 | -1.08594 | 1.10E-09 | 2.43E-08 |
| SULF2 | 29.38604 | 81.53257 | 1.472245 | 6.22E-11 | 2.45E-09 |
| SLAMF9 | 1.172195 | 2.420922 | 1.046345 | 4.72E-05 | 0.000187 |
| COL3A1 | 699.3113 | 1530.681 | 1.130167 | 4.89E-10 | 1.27E-08 |
| NLRP7 | 4.059762 | 24.14938 | 2.572519 | 0.000844 | 0.002263 |
| LRFN2 | 2.178936 | 0.993998 | -1.13231 | 1.71E-06 | 1.13E-05 |
| SERPINB5 | 80.7271 | 161.9297 | 1.004243 | 5.29E-07 | 4.25E-06 |
| BX571818.1 | 11.85725 | 5.697076 | -1.05748 | 0.000269 | 0.000836 |
| EREG | 6.106207 | 24.15619 | 1.984044 | 2.37E-10 | 6.99E-09 |
| CPXM2 | 7.030788 | 14.94256 | 1.087669 | 2.29E-05 | 0.000102 |
| NTRK2 | 1.427381 | 3.454602 | 1.275149 | 0.008318 | 0.016597 |
| PRSS43P | 1.621091 | 0.718637 | -1.17363 | 2.18E-07 | 2.01E-06 |
| HAND2 | 1.343163 | 2.982768 | 1.151017 | 1.06E-05 | 5.30E-05 |
| TCEAL2 | 0.911593 | 2.344857 | 1.363038 | 0.00122 | 0.003126 |
| FAM110B | 1.565968 | 3.154961 | 1.010567 | 4.84E-06 | 2.74E-05 |
| TRIM31 | 48.97779 | 21.7943 | -1.16818 | 0.000516 | 0.001471 |
| PTGDR2 | 1.630741 | 0.702925 | -1.21409 | 0.027535 | 0.046579 |
| LINC02257 | 0.70771 | 2.049252 | 1.533867 | 3.00E-08 | 3.80E-07 |
| CCER2 | 36.90715 | 1.320115 | -4.80517 | 1.31E-09 | 2.79E-08 |
| SPOCD1 | 21.32134 | 9.101352 | -1.22815 | 0.003444 | 0.007676 |
| DES | 266.7863 | 621.5158 | 1.220107 | 2.51E-05 | 0.00011 |
| MKX | 0.632207 | 1.472675 | 1.21997 | 8.76E-07 | 6.45E-06 |
| TCHH | 0.614273 | 2.287565 | 1.89686 | 8.67E-06 | 4.46E-05 |
| AL359715.1 | 1.60643 | 0.659447 | -1.28453 | 4.55E-11 | 1.89E-09 |
| AC004687.1 | 4.134337 | 1.969172 | -1.07007 | 8.06E-05 | 0.000296 |
| PRR33 | 2.628235 | 1.147674 | -1.19538 | 0.009824 | 0.019119 |
| AC091544.4 | 1.61643 | 0.679138 | -1.25103 | 5.06E-08 | 5.92E-07 |
| ADAMTS12 | 3.474437 | 8.281498 | 1.253113 | 9.27E-11 | 3.33E-09 |
| AL135999.3 | 14.35795 | 5.489205 | -1.38718 | 2.97E-06 | 1.80E-05 |
| OSMR | 18.59962 | 37.69645 | 1.019155 | 2.39E-10 | 7.03E-09 |
| KRTAP5-8 | 2.100947 | 0.844943 | -1.31411 | 1.93E-08 | 2.65E-07 |
| CNTNAP2 | 0.655845 | 1.78922 | 1.447904 | 0.000616 | 0.001714 |
| SNX22 | 2.076103 | 0.890681 | -1.2209 | 2.18E-10 | 6.53E-09 |
| HSD17B2 | 17.48842 | 8.115471 | -1.10765 | 0.000244 | 0.000769 |
| TCEAL5 | 0.97948 | 2.232998 | 1.188894 | 0.00032 | 0.000973 |
| AL772337.1 | 3.840719 | 0.87366 | -2.13623 | 3.94E-08 | 4.76E-07 |
| THBS2 | 19.55474 | 41.82451 | 1.09683 | 2.14E-09 | 4.24E-08 |
| MYOSLID | 0.777504 | 2.958168 | 1.927781 | 3.33E-06 | 1.98E-05 |
| WNT11 | 4.463441 | 9.619876 | 1.107862 | 1.59E-05 | 7.45E-05 |
| RNU6-50P | 1.89233 | 0.915608 | -1.04736 | 1.48E-07 | 1.46E-06 |
| DUSP15 | 2.208107 | 0.965739 | -1.19311 | 7.23E-06 | 3.82E-05 |
| KLK10 | 4.891118 | 17.18157 | 1.812626 | 1.53E-07 | 1.49E-06 |
| AC023090.1 | 3.032527 | 1.033968 | -1.55233 | 0.000102 | 0.000365 |
| IBSP | 1.485017 | 10.76634 | 2.857977 | 0.017455 | 0.031371 |
| SPINK1 | 1558.069 | 544.0309 | -1.518 | 3.74E-09 | 6.80E-08 |
| GJB2 | 206.4738 | 523.4695 | 1.342147 | 9.19E-06 | 4.68E-05 |
| THBD | 53.444 | 129.1325 | 1.272752 | 4.87E-08 | 5.73E-07 |
| AC007848.1 | 1.557701 | 0.689143 | -1.17654 | 1.30E-06 | 9.01E-06 |
| RNU2-11P | 2.632246 | 1.248767 | -1.07579 | 8.09E-10 | 1.88E-08 |
| ZNF469 | 1.799328 | 4.297796 | 1.256139 | 1.96E-10 | 5.93E-09 |
| IL36RN | 2.057304 | 8.7911 | 2.095288 | 4.71E-07 | 3.84E-06 |
| ACAN | 1.029459 | 2.08152 | 1.01575 | 0.000502 | 0.00144 |
| BTBD16 | 76.37842 | 30.68292 | -1.31573 | 0.000206 | 0.000665 |
| SELL | 105.8964 | 28.46215 | -1.89554 | 0.004346 | 0.009412 |
| TREML3P | 0.57422 | 1.505069 | 1.390153 | 4.39E-05 | 0.000176 |
| AC112721.1 | 0.347734 | 3.735138 | 3.425107 | 2.77E-06 | 1.69E-05 |
| KRTAP2-3 | 0.452252 | 2.104415 | 2.21822 | 0.019576 | 0.034666 |
| LY6G6C | 6.902638 | 14.67221 | 1.087867 | 0.015575 | 0.028508 |
| PDLIM3 | 6.430234 | 13.59207 | 1.079822 | 7.38E-09 | 1.20E-07 |
| AL355353.1 | 23.00117 | 11.29121 | -1.02651 | 4.50E-13 | 5.14E-11 |
| AC090954.1 | 5.692966 | 1.772595 | -1.68332 | 6.52E-08 | 7.31E-07 |
| CRH | 182.7429 | 71.8658 | -1.34644 | 0.000505 | 0.001445 |
| COMP | 47.71082 | 106.4918 | 1.158353 | 0.001342 | 0.003391 |
| SPP1 | 189.7491 | 679.4251 | 1.840221 | 0.000489 | 0.001406 |
| SRPX | 15.45806 | 38.26401 | 1.307629 | 9.21E-10 | 2.09E-08 |
| KLK7 | 3.195405 | 20.67799 | 2.694025 | 1.47E-07 | 1.45E-06 |
| AC016773.1 | 2.165497 | 1.068244 | -1.01946 | 1.85E-16 | 1.66E-13 |
| KRT6A | 692.0983 | 2087.365 | 1.592634 | 0.000113 | 0.000397 |
| LMNTD2-AS1 | 1.617298 | 0.766189 | -1.07781 | 2.98E-10 | 8.43E-09 |
| VCAN | 16.54606 | 38.96733 | 1.235778 | 4.33E-10 | 1.16E-08 |
| AC010329.1 | 6.543194 | 2.750074 | -1.25052 | 1.72E-06 | 1.14E-05 |
| CPS1 | 0.887685 | 1.822013 | 1.037413 | 0.000516 | 0.001471 |
| IL20RB | 19.61314 | 40.74718 | 1.05488 | 0.007449 | 0.015057 |
| MEG3 | 1.326158 | 3.697098 | 1.479141 | 0.016159 | 0.029397 |
| CHODL | 1.24557 | 2.6179 | 1.071604 | 0.001464 | 0.003663 |
| TNS1 | 14.62903 | 31.20281 | 1.092842 | 2.67E-07 | 2.39E-06 |
| AL008627.1 | 1.637942 | 0.666088 | -1.2981 | 0.001516 | 0.003771 |
| TPPP3 | 14.469 | 29.78936 | 1.041832 | 3.90E-06 | 2.27E-05 |
| DSG1 | 1.287989 | 14.03005 | 3.445329 | 0.016446 | 0.029835 |
| ANPEP | 11.5215 | 43.18701 | 1.906269 | 1.46E-05 | 6.90E-05 |
| GXYLT2 | 2.670766 | 5.746479 | 1.105425 | 8.92E-10 | 2.04E-08 |
| HMGCS2 | 388.3574 | 182.1818 | -1.09201 | 1.20E-05 | 5.89E-05 |
| RPTN | 0.293659 | 1.810892 | 2.624487 | 2.92E-05 | 0.000125 |
| TGM1 | 6.940974 | 32.41554 | 2.223475 | 0.003688 | 0.008136 |
| AL592211.1 | 3.21824 | 1.530246 | -1.07251 | 1.29E-12 | 1.10E-10 |
| ADAMTS16 | 1.13494 | 2.922023 | 1.364352 | 1.42E-05 | 6.78E-05 |
| AC110285.2 | 9.567452 | 3.432677 | -1.4788 | 3.70E-09 | 6.74E-08 |
| C8orf88 | 1.54718 | 3.163359 | 1.031816 | 3.30E-07 | 2.85E-06 |
| COL11A1 | 8.801551 | 19.79357 | 1.169202 | 0.000154 | 0.000517 |
| GOLGA8B | 12.34973 | 5.879294 | -1.07077 | 3.39E-16 | 2.28E-13 |
| GFPT2 | 7.251781 | 15.29567 | 1.076716 | 2.24E-07 | 2.06E-06 |
| GKN1 | 6.343009 | 1.061594 | -2.57894 | 0.000551 | 0.001558 |
| SFTPA2 | 6.127368 | 1.956743 | -1.64681 | 0.023977 | 0.041312 |
| LINC00520 | 0.32528 | 1.657932 | 2.349633 | 1.61E-06 | 1.08E-05 |
| SPTSSB | 97.2237 | 46.97568 | -1.04939 | 0.000879 | 0.002345 |
| ASPG | 1.738609 | 3.581884 | 1.042785 | 0.000561 | 0.00158 |
| LEMD1 | 2.249391 | 5.060397 | 1.169716 | 0.019674 | 0.034806 |
| SLC2A12 | 0.925894 | 2.440784 | 1.398425 | 3.56E-08 | 4.39E-07 |
| MALL | 5.541997 | 13.40438 | 1.274227 | 2.63E-11 | 1.23E-09 |
| ZNF521 | 1.853096 | 4.05125 | 1.128429 | 1.09E-08 | 1.67E-07 |
| PDGFRA | 5.333055 | 10.84666 | 1.024217 | 1.16E-05 | 5.71E-05 |
| GRAMD2A | 1.944046 | 5.548668 | 1.513079 | 0.001131 | 0.002922 |
| AC005291.2 | 1.012954 | 2.301388 | 1.183935 | 6.44E-06 | 3.47E-05 |
| AL138831.3 | 2.444051 | 1.196486 | -1.03047 | 2.12E-12 | 1.56E-10 |
| PACRG | 3.118856 | 1.255451 | -1.31281 | 1.10E-06 | 7.81E-06 |
| MMP3 | 18.37165 | 59.07758 | 1.685129 | 2.70E-08 | 3.48E-07 |
| NUDT11 | 1.60589 | 4.48268 | 1.480988 | 9.38E-07 | 6.82E-06 |
| CKS1BP1 | 1.599263 | 3.680775 | 1.202603 | 1.10E-07 | 1.14E-06 |
| COL14A1 | 8.647207 | 18.127 | 1.067834 | 4.56E-06 | 2.60E-05 |
| ACP7 | 2.042095 | 4.745952 | 1.216648 | 0.001273 | 0.003242 |
| AC107308.1 | 0.626064 | 1.400028 | 1.161074 | 1.53E-05 | 7.17E-05 |
| AC026369.1 | 5.244666 | 2.093294 | -1.32508 | 1.78E-06 | 1.17E-05 |
| KANK4 | 1.729498 | 3.997359 | 1.208694 | 4.41E-10 | 1.18E-08 |
| CNN1 | 74.96835 | 152.843 | 1.027697 | 6.24E-05 | 0.000239 |
| TNC | 31.41547 | 84.08985 | 1.420456 | 2.64E-13 | 3.38E-11 |
| C6orf15 | 1.282806 | 22.44861 | 4.129251 | 0.000853 | 0.002284 |
| ADAM23 | 2.991893 | 6.750077 | 1.173845 | 7.85E-07 | 5.89E-06 |
| MSRB3 | 6.878762 | 14.63082 | 1.08879 | 4.02E-09 | 7.22E-08 |
| GAS1 | 9.410914 | 20.75348 | 1.140947 | 3.31E-08 | 4.12E-07 |
| WNT7A | 4.035802 | 10.28968 | 1.35027 | 1.33E-05 | 6.41E-05 |
| TMEM59L | 0.89893 | 1.848682 | 1.040216 | 3.13E-05 | 0.000132 |
| KEL | 3.325069 | 0.73412 | -2.1793 | 8.99E-07 | 6.59E-06 |
| PAQR6 | 11.53635 | 5.115152 | -1.17334 | 2.85E-12 | 1.96E-10 |
| AC107464.2 | 2.382514 | 1.006525 | -1.2431 | 1.00E-08 | 1.56E-07 |
| SPRR1B | 158.8064 | 634.5176 | 1.998391 | 0.000172 | 0.000571 |
| KRT75 | 3.139006 | 8.437916 | 1.426579 | 0.002617 | 0.006062 |
| MDGA1 | 1.540969 | 3.135856 | 1.025022 | 0.000566 | 0.001592 |
| CALD1 | 47.57408 | 95.82573 | 1.010237 | 1.74E-12 | 1.36E-10 |
| LINC02178 | 1.695477 | 17.44941 | 3.363415 | 0.00084 | 0.002255 |
| AC021491.4 | 1.683286 | 0.718047 | -1.22913 | 6.89E-06 | 3.67E-05 |
| MTND1P23 | 211.753 | 72.15211 | -1.55327 | 0.000402 | 0.001183 |
| GRM3 | 1.626555 | 0.487372 | -1.73872 | 0.000483 | 0.001392 |
| PLPP7 | 0.924857 | 1.910413 | 1.046582 | 1.20E-05 | 5.88E-05 |
| AL390719.2 | 35.18431 | 14.30992 | -1.29792 | 5.71E-16 | 3.30E-13 |
| ACTC1 | 18.19895 | 42.51263 | 1.224036 | 1.42E-05 | 6.78E-05 |
| AC080129.2 | 2.680444 | 1.235464 | -1.11742 | 7.34E-16 | 3.82E-13 |
| ZNF365 | 2.118496 | 4.903835 | 1.21087 | 1.03E-08 | 1.59E-07 |
| CCL7 | 1.482327 | 3.333842 | 1.169322 | 0.000346 | 0.00104 |
| TDRD12 | 1.539159 | 0.725709 | -1.08468 | 0.000429 | 0.00125 |
| MRGPRX3 | 0.117152 | 2.116398 | 4.175154 | 0.004016 | 0.008764 |
| GALNT5 | 2.628591 | 6.77916 | 1.366817 | 1.66E-10 | 5.29E-09 |
| OR7E22P | 2.892736 | 1.278 | -1.17855 | 6.72E-08 | 7.48E-07 |
| ADAMTS15 | 2.704384 | 6.322856 | 1.225276 | 6.56E-06 | 3.52E-05 |
| INHBB | 6.923547 | 14.03651 | 1.019601 | 6.83E-06 | 3.64E-05 |
| IDSP1 | 1.809369 | 0.544664 | -1.73205 | 8.67E-16 | 3.82E-13 |
| LINC02577 | 1.209972 | 2.428243 | 1.00494 | 2.38E-05 | 0.000105 |
| SYNC | 0.865354 | 1.838506 | 1.087171 | 6.60E-12 | 3.98E-10 |
| LRRC15 | 6.000331 | 14.65161 | 1.287945 | 1.85E-06 | 1.21E-05 |
| AL161752.1 | 2.931046 | 1.342653 | -1.12633 | 0.002394 | 0.005613 |
| AC073534.1 | 1.880205 | 0.920229 | -1.03082 | 1.90E-08 | 2.61E-07 |
| MN1 | 1.573312 | 3.902158 | 1.310468 | 4.66E-12 | 2.97E-10 |
| PEG10 | 14.83815 | 35.54187 | 1.260208 | 0.013563 | 0.025284 |
| KRT78 | 1.093183 | 3.795799 | 1.795869 | 0.002343 | 0.005517 |
| VN1R54P | 1.528362 | 0.716251 | -1.09345 | 0.019999 | 0.035253 |
| SORBS1 | 8.258021 | 18.15491 | 1.136492 | 0.000336 | 0.001015 |
| SORCS2 | 2.402831 | 4.823868 | 1.005455 | 1.02E-05 | 5.13E-05 |
| MT1A | 6.987588 | 16.44444 | 1.234733 | 0.013415 | 0.02505 |
| VAT1L | 0.818893 | 1.961035 | 1.259868 | 1.88E-06 | 1.22E-05 |
| PPM1N | 13.25829 | 5.452119 | -1.28201 | 9.67E-07 | 6.99E-06 |
| LINC01871 | 14.26363 | 6.485446 | -1.13706 | 0.018082 | 0.032358 |
| SNAI3 | 3.306349 | 1.641379 | -1.01033 | 0.000348 | 0.001044 |
| FNDC1 | 6.010507 | 13.89314 | 1.208815 | 7.45E-08 | 8.13E-07 |
| PXDN | 13.02349 | 32.58883 | 1.323261 | 1.03E-13 | 1.62E-11 |
| SPRR2F | 3.412983 | 10.89803 | 1.674962 | 0.013715 | 0.025537 |
| FER1L4 | 105.0679 | 43.73522 | -1.26445 | 4.51E-14 | 8.44E-12 |
| PDZD3 | 2.054751 | 0.715709 | -1.52152 | 1.71E-06 | 1.13E-05 |
| ANK1 | 0.70665 | 1.904428 | 1.43029 | 0.001041 | 0.002716 |
| HGF | 0.826451 | 3.827912 | 2.211556 | 1.99E-06 | 1.28E-05 |
| GCNT3 | 1.211626 | 5.427255 | 2.163279 | 0.007573 | 0.015284 |
| DSG3 | 28.041 | 112.5666 | 2.005169 | 8.67E-07 | 6.39E-06 |
| COL6A3 | 58.40933 | 125.0438 | 1.098162 | 1.24E-09 | 2.66E-08 |
| ORM2 | 2.494016 | 1.088588 | -1.19601 | 0.005525 | 0.011584 |
| MYLK | 9.102114 | 18.25414 | 1.00395 | 6.21E-10 | 1.54E-08 |
| LINC02446 | 4.904189 | 1.187715 | -2.04583 | 0.027182 | 0.046101 |
| TBC1D10C | 6.996765 | 3.215098 | -1.12183 | 0.00447 | 0.009649 |
| ARSJ | 2.023415 | 4.619234 | 1.190862 | 3.62E-12 | 2.40E-10 |
| KRT16 | 380.5954 | 958.2632 | 1.332164 | 9.45E-06 | 4.80E-05 |
| CDH17 | 0.524556 | 10.82196 | 4.366721 | 0.00119 | 0.003058 |
| GULP1 | 1.199007 | 2.479921 | 1.048454 | 1.18E-12 | 1.05E-10 |
| PKP1 | 58.26817 | 160.285 | 1.459859 | 0.000566 | 0.001592 |
| PTX3 | 5.585245 | 14.58131 | 1.384428 | 0.000113 | 0.000396 |
| SDR9C7 | 1.964069 | 4.75557 | 1.275772 | 0.00237 | 0.005568 |
| AC018638.7 | 2.773965 | 1.332435 | -1.05788 | 4.28E-12 | 2.75E-10 |
| NKX1-2 | 0.361058 | 1.619968 | 2.165665 | 1.25E-07 | 1.27E-06 |
| AC103691.1 | 2.436375 | 0.936009 | -1.38014 | 8.12E-14 | 1.38E-11 |
| MAP2 | 1.442394 | 3.570774 | 1.307772 | 6.99E-08 | 7.69E-07 |
| KRT14 | 711.3007 | 2390.808 | 1.748967 | 0.005266 | 0.011115 |
| ANXA1 | 189.2836 | 441.5809 | 1.222128 | 8.63E-16 | 3.82E-13 |
| AL161431.1 | 5.481909 | 23.1217 | 2.076497 | 1.51E-08 | 2.17E-07 |
| NKD1 | 1.409326 | 4.790979 | 1.765316 | 2.94E-05 | 0.000125 |
| ACSBG1 | 1.797006 | 0.895404 | -1.00499 | 0.000563 | 0.001586 |
| TSHZ3 | 2.789816 | 5.76989 | 1.048374 | 8.59E-07 | 6.35E-06 |
| GSDMB | 37.52391 | 18.42984 | -1.02577 | 3.07E-13 | 3.79E-11 |
| TESC | 57.20962 | 24.99484 | -1.19463 | 3.70E-10 | 1.02E-08 |
| AC090515.4 | 0.707484 | 1.528057 | 1.110929 | 0.007611 | 0.015343 |
| CST6 | 108.2954 | 242.0256 | 1.160188 | 0.016502 | 0.029914 |
| SPON1 | 10.23645 | 25.65024 | 1.325257 | 2.68E-08 | 3.45E-07 |
| ERVV-2 | 0.598232 | 3.154871 | 2.398805 | 0.01702 | 0.030722 |
| TM4SF1 | 113.8416 | 231.7797 | 1.025726 | 1.10E-10 | 3.79E-09 |
| CXCL5 | 8.237007 | 18.83305 | 1.193074 | 4.73E-05 | 0.000188 |
| CDX1 | 0.760655 | 3.940106 | 2.37292 | 0.000359 | 0.001074 |
| AC010998.3 | 4.774907 | 2.143766 | -1.15532 | 0.000183 | 0.0006 |
| SERPINB2 | 7.376644 | 29.05586 | 1.977793 | 1.60E-09 | 3.33E-08 |
| LINC01833 | 5.643942 | 2.3764 | -1.24793 | 7.75E-07 | 5.83E-06 |
| IL13RA2 | 7.347823 | 19.1022 | 1.37835 | 0.001604 | 0.003957 |
| ZNF683 | 6.271943 | 1.751053 | -1.84069 | 0.002053 | 0.004922 |
| AC133041.1 | 9.164476 | 4.429086 | -1.04904 | 3.28E-07 | 2.83E-06 |
| DSC3 | 35.11308 | 103.0035 | 1.552612 | 0.000224 | 0.000715 |
| IGSF21 | 1.445792 | 3.1849 | 1.139388 | 1.21E-05 | 5.92E-05 |
| AC092329.4 | 2.637443 | 1.295594 | -1.02553 | 2.28E-10 | 6.78E-09 |
| CD109 | 8.166536 | 23.14962 | 1.503192 | 2.49E-17 | 3.09E-14 |
| TF | 2.744191 | 0.3256 | -3.07521 | 0.007631 | 0.015376 |
| TRPS1 | 2.31695 | 4.909998 | 1.083495 | 5.17E-07 | 4.17E-06 |
| POU5F1 | 18.67874 | 6.416221 | -1.5416 | 3.95E-07 | 3.31E-06 |
| AC018809.1 | 3.094006 | 1.42466 | -1.11886 | 1.87E-19 | 6.05E-16 |
| GOLGA8A | 29.5047 | 13.57737 | -1.11974 | 1.19E-12 | 1.05E-10 |
| AL355987.4 | 2.40879 | 0.959599 | -1.32781 | 3.44E-07 | 2.95E-06 |
| AC012307.1 | 8.607263 | 2.392839 | -1.84683 | 1.51E-06 | 1.02E-05 |
| CPED1 | 1.622119 | 3.471407 | 1.097641 | 2.88E-08 | 3.67E-07 |
| AL359881.1 | 3.114241 | 1.354693 | -1.20091 | 0.000249 | 0.000783 |
| RTL9 | 0.047916 | 2.898054 | 5.918431 | 1.52E-06 | 1.03E-05 |
| TFF2 | 32.37845 | 13.00315 | -1.31617 | 0.000217 | 0.000696 |
| CDRT15P9 | 1.628953 | 0.630513 | -1.36935 | 0.000775 | 0.002102 |
| FAM133A | 1.498624 | 3.088769 | 1.043393 | 0.015717 | 0.028726 |
| WNT5B | 8.484361 | 18.38211 | 1.115424 | 7.98E-07 | 5.96E-06 |
| KCNH6 | 7.590407 | 0.106884 | -6.15006 | 0.006584 | 0.01352 |
| PVALB | 47.63095 | 19.06187 | -1.32121 | 0.001746 | 0.004268 |
| DHRS9 | 6.94308 | 14.23127 | 1.035417 | 9.04E-06 | 4.62E-05 |
| CNTN1 | 2.973996 | 10.41252 | 1.807845 | 3.79E-07 | 3.20E-06 |
| GPR1 | 1.201478 | 3.564413 | 1.568855 | 1.27E-07 | 1.29E-06 |
| CTSE | 87.08388 | 31.37948 | -1.47258 | 0.00034 | 0.001026 |
| SPINK4 | 23.94038 | 8.949144 | -1.41962 | 0.00209 | 0.004999 |
| AC083967.1 | 0.212505 | 4.251659 | 4.32246 | 0.002951 | 0.006733 |
| SGCD | 1.023917 | 2.240801 | 1.129916 | 1.31E-08 | 1.94E-07 |
| AL353572.4 | 5.184672 | 2.5495 | -1.02404 | 4.89E-06 | 2.76E-05 |
| LOX | 9.461418 | 20.03212 | 1.082187 | 1.31E-11 | 6.87E-10 |
| AL139123.1 | 2.171667 | 1.070839 | -1.02006 | 1.56E-13 | 2.26E-11 |
| ITGBL1 | 1.812199 | 4.163123 | 1.199925 | 2.35E-06 | 1.48E-05 |
| CHRDL2 | 8.992424 | 18.97188 | 1.07708 | 1.65E-07 | 1.59E-06 |
| GBP6 | 6.034408 | 14.52536 | 1.267289 | 0.000124 | 0.000429 |
| AC027348.1 | 3.034776 | 1.111642 | -1.4489 | 8.12E-11 | 3.01E-09 |
| NRG1 | 0.576381 | 2.501685 | 2.117806 | 6.63E-08 | 7.40E-07 |
| ADAMTS9 | 2.20815 | 4.564853 | 1.04773 | 1.61E-06 | 1.08E-05 |
| TNXB | 1.433643 | 3.056063 | 1.091989 | 5.65E-05 | 0.00022 |
| NRP2 | 6.118521 | 13.68556 | 1.161399 | 5.49E-13 | 5.85E-11 |
| COL8A1 | 10.16878 | 20.71116 | 1.026261 | 4.94E-08 | 5.80E-07 |
| AC084880.1 | 3.059845 | 6.921388 | 1.177603 | 4.77E-07 | 3.89E-06 |
| KRT16P4 | 0.585978 | 1.405662 | 1.262332 | 0.016365 | 0.029709 |
| TNFRSF14-AS1 | 3.964015 | 1.549309 | -1.35534 | 1.72E-12 | 1.36E-10 |
| TMPRSS11A | 1.418655 | 3.756681 | 1.404935 | 0.029578 | 0.049567 |
| KLK6 | 6.810029 | 37.65335 | 2.467045 | 3.52E-09 | 6.47E-08 |
| TENM3 | 1.14727 | 3.615232 | 1.655884 | 2.85E-10 | 8.13E-09 |
| AADACP1 | 1.379176 | 2.980101 | 1.111554 | 0.000231 | 0.000735 |
| LAMC2 | 99.30876 | 247.6727 | 1.318442 | 4.94E-06 | 2.78E-05 |
| S100A7A | 2.973371 | 16.50878 | 2.473063 | 0.006823 | 0.013932 |
| FHL1 | 15.17662 | 33.04348 | 1.122515 | 1.08E-06 | 7.70E-06 |
| PCDH18 | 2.627186 | 5.632528 | 1.100264 | 1.08E-07 | 1.12E-06 |
| AL355607.1 | 1.049061 | 2.267522 | 1.112017 | 0.001833 | 0.004456 |
| CASQ2 | 3.91569 | 10.18208 | 1.378694 | 2.24E-05 | 9.97E-05 |
| TRGC2 | 3.666424 | 1.498573 | -1.29078 | 0.027143 | 0.046045 |
| AC010329.5 | 2.628993 | 0.624476 | -2.07379 | 7.05E-06 | 3.74E-05 |
| FGFBP1 | 58.34184 | 185.8498 | 1.671534 | 6.53E-07 | 5.03E-06 |
| CCL15 | 2.150633 | 0.94104 | -1.19243 | 2.65E-06 | 1.63E-05 |
| KLK12 | 0.714855 | 2.08692 | 1.545653 | 0.006553 | 0.013461 |
| PDE3A | 0.809807 | 1.710169 | 1.078488 | 3.54E-09 | 6.50E-08 |
| AREG | 55.4321 | 113.8094 | 1.037826 | 8.78E-07 | 6.46E-06 |
| AC008759.2 | 3.499273 | 1.229297 | -1.50922 | 1.83E-10 | 5.64E-09 |
| COL1A2 | 456.0737 | 951.5673 | 1.061039 | 1.09E-09 | 2.42E-08 |
| HPSE2 | 1.627972 | 3.908893 | 1.263684 | 1.29E-05 | 6.26E-05 |
| TFF1 | 151.7192 | 63.72638 | -1.25144 | 0.000733 | 0.002 |
| SCEL | 5.155043 | 12.84433 | 1.317076 | 1.50E-08 | 2.16E-07 |
| HSPB8 | 34.67943 | 72.98882 | 1.073596 | 3.67E-07 | 3.12E-06 |
| HRH3 | 2.058294 | 0.731495 | -1.49253 | 0.00011 | 0.000386 |
| TSPAN7 | 7.041167 | 20.88895 | 1.568854 | 0.009197 | 0.018066 |
| CRYAB | 9.834575 | 21.94998 | 1.158285 | 1.16E-09 | 2.53E-08 |
| UGT2B15 | 14.7817 | 3.207583 | -2.20425 | 3.84E-05 | 0.000157 |
| AL133330.1 | 0.740654 | 1.506163 | 1.024005 | 7.44E-05 | 0.000277 |
| AC040174.2 | 1.920227 | 0.799305 | -1.26446 | 0.000394 | 0.001162 |
| PTGIS | 12.13616 | 28.91642 | 1.252577 | 8.23E-06 | 4.27E-05 |
| AC012354.2 | 18.23721 | 5.175026 | -1.81725 | 1.66E-07 | 1.60E-06 |
| SLC10A6 | 1.644663 | 3.835886 | 1.221768 | 5.93E-07 | 4.66E-06 |
| KRTAP5-9 | 8.480225 | 3.35191 | -1.33912 | 3.33E-08 | 4.14E-07 |
| AC010329.2 | 3.084118 | 0.528213 | -2.54567 | 5.87E-09 | 9.90E-08 |
| ABCA12 | 1.931625 | 5.254269 | 1.443675 | 3.86E-11 | 1.65E-09 |
| PRKG1 | 0.715469 | 1.583205 | 1.145888 | 6.36E-11 | 2.50E-09 |
| EDAR | 1.458666 | 3.089023 | 1.082501 | 0.000169 | 0.000562 |
| HYAL1 | 2.478408 | 5.232703 | 1.078143 | 5.33E-05 | 0.000209 |
| SCG2 | 2.760135 | 5.79889 | 1.071038 | 3.21E-07 | 2.78E-06 |
| MYADM | 27.47653 | 61.13442 | 1.153785 | 1.54E-11 | 7.78E-10 |
| KLK8 | 0.852629 | 6.111456 | 2.841526 | 2.20E-07 | 2.03E-06 |
| GLI2 | 0.82194 | 2.052445 | 1.320239 | 4.38E-09 | 7.74E-08 |
| SERPINB7 | 3.014145 | 12.7193 | 2.077199 | 6.40E-08 | 7.20E-07 |
| CYP4F12 | 31.31316 | 13.06153 | -1.26145 | 2.26E-08 | 3.03E-07 |
| SCNN1G | 60.34658 | 22.13512 | -1.44693 | 3.95E-07 | 3.31E-06 |
| ITGA11 | 5.399171 | 13.99803 | 1.374414 | 4.36E-08 | 5.20E-07 |
| SCN4B | 1.122401 | 2.36101 | 1.072816 | 2.39E-07 | 2.17E-06 |
| EFEMP1 | 35.04161 | 70.39421 | 1.006388 | 1.28E-10 | 4.28E-09 |
| AP003419.3 | 3.992614 | 1.886893 | -1.08132 | 2.04E-12 | 1.52E-10 |
| MANCR | 0.592126 | 2.907552 | 2.29583 | 1.27E-07 | 1.28E-06 |
| ACTG2 | 122.0538 | 281.2811 | 1.204495 | 2.99E-05 | 0.000127 |
| CD8B | 3.756476 | 1.555789 | -1.27173 | 0.001723 | 0.004218 |
| NGF | 1.772249 | 3.947659 | 1.155416 | 6.75E-09 | 1.11E-07 |
| TLL1 | 0.826233 | 2.003646 | 1.278008 | 3.54E-10 | 9.80E-09 |
| SOST | 2.41268 | 13.93645 | 2.530155 | 0.005852 | 0.012192 |
| BDKRB1 | 1.770921 | 3.559044 | 1.00699 | 2.77E-07 | 2.46E-06 |
| STK32A-AS1 | 4.096092 | 1.627046 | -1.33199 | 0.003473 | 0.007733 |
| CAV1 | 87.17646 | 207.7161 | 1.252602 | 5.58E-12 | 3.46E-10 |
| PCP2 | 5.201939 | 2.290213 | -1.18357 | 6.38E-14 | 1.15E-11 |
| DCHS1 | 2.910771 | 5.912272 | 1.022311 | 5.36E-08 | 6.22E-07 |
| CCDC80 | 13.23514 | 29.528 | 1.157711 | 5.72E-10 | 1.44E-08 |
| AC012354.7 | 4.678363 | 1.544915 | -1.59848 | 3.03E-05 | 0.000129 |
| AC112721.2 | 0.565722 | 4.109778 | 2.860896 | 6.43E-09 | 1.07E-07 |
| TEKT5 | 4.244192 | 1.958434 | -1.11579 | 9.31E-06 | 4.74E-05 |
| AP000867.5 | 2.487147 | 0.880444 | -1.49819 | 1.10E-07 | 1.14E-06 |
| IL24 | 2.458141 | 5.341307 | 1.119625 | 5.08E-06 | 2.85E-05 |
| FIBCD1 | 1.323619 | 3.591803 | 1.44022 | 0.004405 | 0.009528 |
| NPBWR1 | 1.323594 | 3.428441 | 1.373092 | 0.001249 | 0.003189 |
| CYTL1 | 2.42043 | 8.808225 | 1.863588 | 6.94E-07 | 5.30E-06 |
| RASSF9 | 0.985017 | 2.202256 | 1.160762 | 2.02E-09 | 4.04E-08 |
| MIR141 | 1.699048 | 0.448019 | -1.92309 | 1.06E-10 | 3.68E-09 |
| KRT1 | 14.17442 | 190.6193 | 3.749333 | 0.000502 | 0.001438 |
| TBX4 | 1.340111 | 2.999652 | 1.162443 | 0.001232 | 0.003153 |
| AL158063.1 | 1.480905 | 0.695032 | -1.09133 | 1.48E-14 | 3.42E-12 |
| GZMM | 7.182494 | 3.301438 | -1.12139 | 0.007602 | 0.015331 |
| EGFR | 32.67782 | 84.75615 | 1.375006 | 9.27E-11 | 3.33E-09 |
| ACSM6 | 4.8133 | 2.262623 | -1.08903 | 3.93E-07 | 3.30E-06 |
| MOGAT2 | 5.940507 | 1.944648 | -1.61108 | 1.86E-07 | 1.76E-06 |
| AL121829.2 | 1.901288 | 0.950179 | -1.00071 | 6.33E-10 | 1.56E-08 |
| CDH16 | 0.418108 | 1.693435 | 2.018003 | 0.002332 | 0.005493 |
| AL390294.1 | 1.568926 | 0.640249 | -1.29307 | 1.14E-09 | 2.50E-08 |
| EMP1 | 37.10308 | 86.36324 | 1.218878 | 1.16E-15 | 4.68E-13 |
| COL2A1 | 0.607138 | 4.851953 | 2.998469 | 0.003664 | 0.008087 |
| PGLYRP3 | 3.957366 | 13.28923 | 1.747645 | 0.009152 | 0.017995 |
| CEMIP | 5.645066 | 14.09766 | 1.320393 | 2.33E-06 | 1.47E-05 |
| CPXM1 | 30.08809 | 62.84271 | 1.062553 | 1.69E-05 | 7.84E-05 |
| IFITM3P9 | 0.878399 | 2.293181 | 1.384401 | 0.021423 | 0.037393 |
| RBP1 | 19.87845 | 41.07808 | 1.047164 | 5.47E-08 | 6.33E-07 |
| KRT79 | 5.641663 | 12.38642 | 1.134567 | 0.000228 | 0.000725 |
| THBS1 | 62.86591 | 149.723 | 1.251946 | 4.88E-14 | 8.87E-12 |
| U47924.1 | 1.577195 | 0.765594 | -1.04271 | 1.20E-13 | 1.80E-11 |
| SOX7 | 5.530713 | 12.34134 | 1.157961 | 3.04E-10 | 8.58E-09 |
| AC068594.1 | 1.462595 | 0.566337 | -1.3688 | 1.55E-07 | 1.51E-06 |
| AL096855.1 | 2.550395 | 1.022402 | -1.31876 | 3.06E-08 | 3.86E-07 |
| IL1B | 18.95838 | 38.60068 | 1.02579 | 0.000381 | 0.001131 |
| LINC02154 | 2.275104 | 17.4 | 2.935083 | 0.007329 | 0.014841 |
| SLC47A2 | 0.288136 | 2.349059 | 3.027263 | 0.014769 | 0.027202 |
| AC010636.1 | 2.927154 | 1.314839 | -1.15461 | 0.000149 | 0.000505 |
| MIR200A | 10.76561 | 4.79014 | -1.16829 | 9.16E-08 | 9.68E-07 |
| F2RL2 | 1.691848 | 4.742591 | 1.487075 | 1.44E-06 | 9.84E-06 |
| MMP13 | 27.29817 | 85.51763 | 1.647418 | 3.73E-06 | 2.19E-05 |
| PPP2R2C | 2.060449 | 4.739798 | 1.201866 | 0.009382 | 0.018364 |
| AC053503.3 | 1.905755 | 3.900452 | 1.033278 | 0.004522 | 0.009746 |
| CLSTN2 | 0.552746 | 2.34715 | 2.086221 | 9.33E-08 | 9.80E-07 |
| SYNDIG1 | 2.195533 | 5.389607 | 1.295609 | 1.66E-07 | 1.60E-06 |
| AP001189.3 | 0.762558 | 1.60676 | 1.075235 | 2.11E-09 | 4.18E-08 |
| LINC00967 | 13.2052 | 5.836959 | -1.17782 | 0.000582 | 0.001629 |
| INHBA | 10.53963 | 23.41448 | 1.151576 | 3.40E-12 | 2.26E-10 |
| RAB26 | 5.92679 | 2.857451 | -1.05252 | 3.93E-05 | 0.00016 |
| AL359715.2 | 1.746114 | 0.669786 | -1.38237 | 1.72E-12 | 1.36E-10 |
| FAP | 4.129666 | 8.766098 | 1.08591 | 1.12E-10 | 3.82E-09 |
| CD96 | 11.4703 | 4.857064 | -1.23975 | 8.39E-14 | 1.40E-11 |
| SLC5A1 | 0.624093 | 1.56597 | 1.327224 | 0.000688 | 0.00189 |
| BCHE | 1.455311 | 4.43008 | 1.606005 | 1.54E-08 | 2.20E-07 |
| HES6 | 40.23603 | 17.75197 | -1.18051 | 0.00244 | 0.005709 |
| STC1 | 16.70594 | 36.65216 | 1.133537 | 9.26E-10 | 2.10E-08 |
| SH3RF2 | 3.59222 | 7.302482 | 1.023511 | 6.24E-05 | 0.000239 |
| ASB5 | 0.604251 | 1.993101 | 1.721795 | 0.000699 | 0.001919 |
| PLAU | 118.1843 | 255.4457 | 1.111978 | 2.96E-13 | 3.71E-11 |
| NOG | 0.916393 | 4.347653 | 2.246199 | 0.025218 | 0.04316 |
| HTR7 | 2.698135 | 5.643663 | 1.064669 | 5.96E-05 | 0.000229 |
| FLNC | 10.37316 | 27.95083 | 1.430035 | 1.25E-08 | 1.88E-07 |
| LEAP2 | 19.37514 | 4.154021 | -2.22163 | 2.90E-10 | 8.23E-09 |
| KITLG | 8.348202 | 17.87885 | 1.098717 | 5.22E-10 | 1.35E-08 |
| DAB1 | 4.241533 | 1.85011 | -1.19698 | 0.000488 | 0.001405 |
| ANKRD44-AS1 | 2.37162 | 0.811051 | -1.54801 | 6.38E-06 | 3.45E-05 |
| CTSV | 8.665109 | 22.85324 | 1.399109 | 6.77E-08 | 7.51E-07 |
| SLC22A3 | 2.752171 | 6.59684 | 1.261205 | 2.71E-09 | 5.17E-08 |
| OVGP1 | 9.879559 | 4.39665 | -1.16804 | 2.20E-09 | 4.34E-08 |
| COLQ | 1.54382 | 0.766735 | -1.0097 | 1.40E-11 | 7.19E-10 |
| ITGA5 | 43.7623 | 89.91554 | 1.038882 | 5.48E-11 | 2.22E-09 |
| AC121757.1 | 0.799399 | 1.70351 | 1.091523 | 7.91E-06 | 4.12E-05 |
| NUDT10 | 0.562682 | 1.581656 | 1.491043 | 5.12E-06 | 2.87E-05 |
| LINC02544 | 1.49567 | 3.823764 | 1.354201 | 2.50E-06 | 1.55E-05 |
| SYT8 | 132.0286 | 65.59526 | -1.00919 | 0.024204 | 0.041644 |
| RNASE7 | 5.607706 | 16.30328 | 1.539679 | 0.000224 | 0.000715 |
| HMGA2 | 2.483681 | 7.045094 | 1.504139 | 5.58E-10 | 1.42E-08 |
| BOC | 2.001835 | 4.976554 | 1.313824 | 7.54E-08 | 8.22E-07 |
| RPSAP52 | 0.557895 | 1.6709 | 1.58256 | 4.81E-09 | 8.36E-08 |
| EFCAB1 | 0.810054 | 1.778868 | 1.134869 | 0.002116 | 0.005048 |
| SBSN | 32.66979 | 106.4443 | 1.704069 | 0.005392 | 0.011341 |
| ALDH1L2 | 1.695616 | 3.564993 | 1.07209 | 4.26E-11 | 1.78E-09 |
| MIR429 | 31.13443 | 12.65657 | -1.29862 | 2.14E-10 | 6.42E-09 |
| TPPP | 1.754836 | 3.626272 | 1.047151 | 1.99E-05 | 9.01E-05 |
| CYP4F29P | 10.94096 | 4.047438 | -1.43466 | 0.000204 | 0.000661 |
| SHISAL1 | 1.02509 | 2.359411 | 1.202677 | 4.95E-10 | 1.29E-08 |
| TMEM178A | 4.056014 | 1.782533 | -1.18613 | 2.74E-10 | 7.81E-09 |
| PSORS1C3 | 16.65932 | 7.519467 | -1.14763 | 0.005977 | 0.012432 |
| AL121761.1 | 1.472476 | 2.985228 | 1.019597 | 0.003499 | 0.007782 |
| DSP | 116.5196 | 283.8783 | 1.2847 | 1.71E-10 | 5.41E-09 |
| AL450384.2 | 7.138498 | 2.982307 | -1.25919 | 8.20E-13 | 7.76E-11 |
| COL17A1 | 50.17466 | 106.4609 | 1.085293 | 0.00137 | 0.003454 |
| KRTAP5-10 | 4.236896 | 1.408768 | -1.58857 | 9.76E-11 | 3.47E-09 |
| STEAP1B | 0.592244 | 1.42283 | 1.2645 | 8.37E-07 | 6.22E-06 |
| CYP4F8 | 47.11746 | 16.45085 | -1.5181 | 1.92E-06 | 1.24E-05 |
| OGN | 2.027574 | 4.329089 | 1.094309 | 0.000141 | 0.00048 |
| CSF3 | 6.765796 | 14.53136 | 1.102838 | 0.002043 | 0.004904 |
| SH2D5 | 0.981772 | 2.582184 | 1.395133 | 2.87E-08 | 3.66E-07 |
| PLPPR1 | 1.57675 | 0.607685 | -1.37556 | 0.018444 | 0.032884 |
| TTR | 31.9444 | 3.296783 | -3.27643 | 7.38E-06 | 3.88E-05 |
| WFDC12 | 0.520467 | 3.833581 | 2.880815 | 0.023692 | 0.040876 |
| ADAM19 | 11.89416 | 24.39699 | 1.03645 | 2.58E-06 | 1.59E-05 |
